# TEVSER: a theory of evolving self-representations

**DOI:** 10.3389/fnhum.2026.1858621

**Published:** 2026-07-09

**Authors:** Igor Pivovarov

**Affiliations:** Moscow Institute of Physics and Technology, Dolgoprudny, Russia

**Keywords:** artificial agents, consciousness, evolution, intelligence, learning, predictive processing, self-organization, self-representation

## Abstract

This paper proposes TEVSER (Theory of Evolving Self-Representations), a framework describing how increasingly complex forms of regulation give rise to psyche, consciousness, and intelligence. The central idea is that a living system is a self-regulating system that maintains homeostasis. Within this perspective, regulation can be described as a hierarchy of self-representations (*Ω*), emerging as control structures that guide behavior. Within this hierarchy, distinct functional levels correspond to qualitatively different forms of cognition. In particular, the framework identifies the emergence of a phenomenological internal world (Ω^2^), spatial subjectivity (“here,” Ω^3^), temporal presence (“now,” Ω^6^), behavioral intelligence (Ω^8^), self-consciousness (“who,” Ω^10^), and abstract symbolic intellect (Ω^11^). Within this perspective, consciousness is not treated as a singular entity but as a structured and graded property arising from the organization of self-representing systems. The framework offers a constructive approach to the hard problem of consciousness, addressing the apparent paradox between the material nature of the brain and the seemingly immaterial character of subjective experience. TEVSER integrates and extends existing approaches, including predictive coding, active inference, higher-order theories, and integrated information theory, by situating them within a unified hierarchical architecture. Importantly, the framework generates a set of testable hypotheses linking levels of self-representation to neural organization, behavior, and evolutionary complexity. These predictions provide a basis for empirical validation and position TEVSER not only as a conceptual model but as a research program for investigating consciousness and intelligence in biological and artificial systems.

## 1 Introduction

Understanding how consciousness, subjectivity, and intelligence emerge from physical systems remains a central challenge across neuroscience, cognitive science, and artificial intelligence. Despite extensive research, these concepts are often defined inconsistently and used interchangeably. Terms such as psyche, consciousness, self-consciousness, and subject frequently lack clear operational definitions, leading to ambiguity and fragmentation across disciplines.

A major source of this difficulty is that many existing approaches rely either on phenomenological descriptions or on high-level computational abstractions, without providing a constructive account of how these phenomena arise from underlying system dynamics. As a result, it remains unclear how to systematically relate observable behavior, internal representations, and subjective experience within a unified framework.

Several influential theories have addressed different aspects of this problem. For example, predictive processing and active inference frameworks model cognition as hierarchical inference, while integrated information theory focuses on the structural properties of conscious systems. Other approaches, such as global workspace and higher-order theories, emphasize specific functional or architectural features associated with conscious processing. While each of these frameworks captures important elements, none provides a fully constructive, evolutionarily grounded account that unifies regulation, representation, and subjectivity within a single formal perspective (Anokhin, 2021).

In this work, we propose the Theory of Evolving Self-Representations (TEVSER), a framework that approaches consciousness and intelligence from the standpoint of self-regulating systems. The central assumption is that any living system maintaining homeostasis must include regulatory mechanisms, and that increasingly complex forms of cognition emerge through the progressive integration and coordination of these regulators.

Within this perspective, we introduce the concept of self-representation as the internal structure formed by the models underlying regulatory processes. Building on this idea, we propose a hierarchical organization of self-representations, in which each level corresponds to a qualitatively new form of regulation. This hierarchy spans from simple, independent regulators to integrated systems capable of spatial self-modeling, learning, attention, and abstract cognition.

A key feature of this approach is that it provides a constructive reinterpretation of core concepts. In particular, psyche is understood as a system of differentiated internal signals, subjectivity as the emergence of a spatially localized perspective, and consciousness as the dynamic selection and prioritization of representations within a unified internal space. These definitions are grounded in functional roles and can, in principle, be operationalized and tested.

By framing cognition and consciousness as properties of evolving regulatory architectures, TEVSER offers a unified perspective that is compatible with, but distinct from, existing theories. It also suggests a set of testable predictions regarding the relationship between hierarchical organization, attention, learning, and the emergence of conscious processing, with implications for both neuroscience and the design of artificial systems.

## 2 Theoretical foundations

### 2.1 Systems, regulation, and self-representation

TEVSER begins from the assumption that living organisms can be understood as *self-regulating* systems that maintain *homeostasis* under changing external conditions. In both biological and artificial contexts, such systems preserve a set of internal variables within viable ranges through regulatory processes. From this perspective, cognition and consciousness should not be treated as isolated phenomena, but as higher-order developments of regulation.

A central theoretical basis for this approach is the Good Regulator Theorem (Conant and Ashby, 1970), according to which every good regulator of a system must contain a *model of that system*. In the present framework, this principle is generalized to living systems maintaining homeostasis through multiple interacting regulators. Even the simplest regulator must, in some form, embody the variable or subsystem it regulates.

Let S denote a system maintaining homeostasis, and let R_i_ denote regulators acting on subsystems S_i_ ⊆ S. Each regulator R_i_ is associated with a model of the corresponding subsystem, denoted ρ_i_(S_i_). The complete set of such models constitutes the system’s self-representation.

Thus, following the original definition of TEVSER, the *self-representation* of a system S is denoted by *Ω* and defined as the complete set of models of all its regulators. In the simplest case, when regulators operate independently, *Ω* consists of independent models. When regulators become interdependent, higher-order regulatory organization emerges, and *Ω* acquires increasing internal structure.

Importantly, self-representation here is not an external mathematical description imposed by an observer. A representation of a system may exist for an external observer, but self-representation *Ω* *exists within the system itself* for the purpose of self-regulation. It is an internal functional organization arising from the maintenance of homeostasis.

### 2.2 Evolutionary and constructive approach

The TEVSER framework is explicitly evolutionary. It assumes that the forms of self-representation observed in complex organisms did not appear all at once, but emerged gradually as new regulatory structures conferred survival advantages. In this view, the evolution of life can be interpreted as the evolution of regulators, from primitive error-based regulation to increasingly integrated systems capable of modeling both the environment and the organism itself.

This evolutionary perspective is not merely historical background. It plays a methodological role in the construction of the theory. TEVSER assumes that a new functional entity can emerge and persist only if it provides an advantage in maintaining homeostasis under relevant environmental conditions. Therefore, each new level of self-representation must correspond to a new regulatory capability with functional consequences.

This leads to a constructive approach. Definitions in the theory are introduced not on the basis of intuition alone, but on the basis of function. A new concept is justified only if it corresponds to a distinct organizational or regulatory role that can, at least in principle, be identified, instantiated, or indirectly verified.

Several general principles follow from this approach:

#### 2.2.1 Evolutionary advantage principle

A new regulatory structure is preserved only if it improves the organism’s capacity to maintain homeostasis, adapt to changing conditions, or survive long enough to reproduce.

#### 2.2.2 Energy efficiency principle

Any new regulatory function entails energetic cost. Over evolutionary timescales, only those regulatory architectures that can be energetically sustained remain stable.

#### 2.2.3 Constructive design principle

Theoretical entities should correspond to functionally distinct structures or processes. In this sense, the theory aims not only to describe consciousness, but also to provide a framework for constructing systems with progressively richer forms of self-representation.

#### 2.2.4 Survivorship principle

What is observed in biology is not the full space of possible forms, but only those configurations that remained viable over time. This principle is important when reconstructing the likely stages in the evolution of self-representation.

Together, these principles justify treating the hierarchy of self-representation not as an arbitrary classification, but as an evolutionary and functional sequence.

### 2.3 Phylogenesis and ontogenesis

Evolution of self-representations unfolds across two distinct but related processes: phylogenesis and ontogenesis.

*Phylogenesis* refers to the emergence of new forms of self-representation across species evolution through structural and physiological changes. In this domain, new regulatory architectures appear and are stabilized over evolutionary time because they confer adaptive advantages.

*Ontogenesis* refers to the development of self-representation within the lifetime of an individual organism. Some forms of self-representation are phylogenetically inherited as capacities, but become fully realized only during development through maturation, learning, and interaction with the environment.

This distinction is essential because not all levels of *Ω* arise in the same way. Earlier levels are likely rooted primarily in phylogenetic evolution, where regulatory architectures become biologically instantiated. Higher levels, especially those involving learning, abstract cognition, and socially mediated forms of self-representation, depend increasingly on ontogenetic development.

Thus, TEVSER does not treat the hierarchy of self-representation as a purely evolutionary ladder fixed once and for all at the species level. Rather, it proposes that phylogenesis builds the structural possibility of a level, while ontogenesis may realize, refine, or extend it within the individual.

This dual perspective also helps explain why later forms of consciousness and selfhood cannot be inferred solely from anatomy. They depend not only on inherited regulatory architecture, but also on developmental processes that reorganize and enrich *Ω* over time.

### 2.4 Hierarchical development of self-representation

Within this framework, TEVSER proposes that self-representation develops hierarchically. Each level of *Ω* corresponds to a new form of organization among regulatory models. Lower levels involve independent or weakly coordinated regulators, whereas higher levels involve integration, differentiation, learning, selection, and increasingly complex relations between the organism and its world.

A new level is introduced only when a genuinely new regulatory function appears. This is important because the hierarchy is not intended as a descriptive inventory of traits, but as a sequence of constructive transitions. Each level marks a qualitative change in what the system can do in order to preserve itself.

Such transitions may include, for example, the coordination of previously independent regulators, the differentiation of signals within a common internal space, the emergence of spatial self-localization, the capacity to form new state connections through learning, or the selective prioritization of signals through attention.

In this sense, the hierarchy of *Ω* is both a model of biological evolution and a functional map of increasing cognitive complexity. It provides the bridge from primitive regulation to psyche, subjectivity, consciousness, and higher intelligence.

### 2.5 From regulation to cognition

The central theoretical move of TEVSER is to treat cognition as an advanced form of regulation rather than as a fundamentally separate domain. On this view, perception, learning, attention, and conscious processing emerge when regulatory models become sufficiently integrated and differentiated within a single evolving self-representation *Ω*.

This approach allows TEVSER to reinterpret traditional cognitive concepts in operational terms. Psyche can be understood as a structured internal domain of differentiated sensations and emotions. Subjectivity emerges when the organism acquires a localized perspective within its own representational space. Consciousness emerges when the system can dynamically select and prioritize parts of *Ω* in accordance with current regulatory demands.

Thus, TEVSER frames consciousness not as a primitive essence, but as a later and more complex stage in the development of self-representation. This preserves continuity between basic life processes and higher cognition, while still allowing qualitative transitions between levels.

## 3 TEVSER: hierarchy of self-representations

### 3.1 Overview of the hierarchical model

Within the TEVSER framework, the evolution of cognition is described as a sequence of transitions between qualitatively distinct forms of self-representation, denoted by *Ω*.

Each level corresponds to a new organizational principle that enhances the system’s ability to maintain homeostasis under increasingly complex environmental conditions. These levels are not arbitrary classifications, but constructive stages: each emerges as a solution to a limitation of the previous one.

Lower levels are primarily shaped by phylogenesis, while higher levels increasingly depend on ontogenetic development. The hierarchy spans from independent regulators to systems capable of attention, learning, spatial self-representation, and temporally extended experience.

The *Ω* hierarchy is primarily an analytical model of increasing regulatory complexity – a functional sequence of qualitative transitions. The evolutionary datings and taxonomic examples used throughout are best understood as candidate instantiations of the functional levels – grounded in biological evidence, but approximate and subject to revision as the evidence develops.

In biological reality, transitions between levels are likely gradual, overlapping, and potentially convergent across lineages; the same *Ω* level may have been reached independently by different clades.

The term “self-representation” carries different weight at different levels: at lower levels (Ω^0^ to Ω^1^) it denotes implicit regulatory organization without phenomenological content; phenomenological content is explicitly introduced only from Ω^2^ onward, as motivated by the emergence of a differentiated internal signal space.

### 3.2 Pre-neural level: emergence of self-representation

#### 3.2.1 Emergence of regulation

Before introducing the hierarchy of self-representations, it is necessary to clarify how the first regulatory structures emerge. In TEVSER, this transition marks the boundary between non-living and living systems.

In a pre-biotic environment, a vast number of molecular configurations continuously form and decay. Some configurations are more stable than others and therefore persist longer. As a result, an external observer is more likely to encounter relatively stable structures, even though the majority of possible configurations are short-lived. However, stability alone does not constitute life.

A crucial step occurs when a *boundary* emerges that separates an internal region from the external environment. Once such a boundary exists, the internal state of the system becomes, in general, different from the external state. This immediately creates a non-equilibrium condition: physical processes tend to eliminate this difference, driving the system toward equilibrium.

Any bounded system with a distinct internal composition is therefore inherently unstable. Without additional mechanisms, such a system will either dissolve or collapse due to processes such as diffusion and osmotic imbalance. The maintenance of internal structure requires active processes that counteract these tendencies. As emphasized by Erwin Schrödinger (Schrödinger, 1944), living systems are characterized not by equilibrium, but by the continuous maintenance of a stationary state through the expenditure of energy.

A qualitatively new situation arises when processes appear that maintain internal variables within certain ranges. At this point, the system begins to exhibit regulation. These processes need not be precise or optimized; even simple or partially effective mechanisms can significantly increase the lifetime of the system compared to purely passive structures. This kind of regulation, keeping basic homeostasis of a boundary within an environment is understood in detail in modern biophysics (Ataullakhanov et al., 2009).

From the perspective of TEVSER, the emergence of such processes corresponds to the appearance of the *first regulator*. The regulator does not “aim” to preserve the system in any intentional sense. Rather, systems that happen to include such mechanisms *persist longer* and are therefore more likely to be observed.

This marks the transition from merely stable structures to systems that maintain a stationary state—that is, systems that actively preserve their internal organization over time. In this sense, life can be understood not as the presence of a particular molecule or structure, but as the emergence of regulation maintaining homeostasis.

This distinction is important in relation to the concept of *replicators*. Molecules capable of replication, such as nucleic acids, can persist and reproduce under certain conditions, which is sometimes addressed as a proper basis for life (Dawkins, 1976). However, replication alone does not ensure the maintenance of a system’s internal state. A collection of replicators without regulatory processes does not constitute a self-preserving system. In contrast, even a very simple regulatory mechanism that maintains internal stability can serve as the basis for life.

Thus, in TEVSER, the defining feature of living systems is not replication, but the presence of regulatory processes that maintain homeostasis.

This transition likely occurred relatively early in the history of life on Earth, on the order of hundreds of millions of years after planetary formation, when the first stable homeostatic structures emerged. While the exact mechanisms remain uncertain, the key point is that the emergence of regulation marks a qualitative shift: from passive stability to active maintenance of internal structure.

#### 3.2.2 Independent regulators and Ω^0^

Once regulatory processes emerge, the system can be described as consisting of a set of regulators R_i_, each maintaining a particular variable or subsystem S_i_. Each regulator is associated with a model ρ_i_(S_i_), in accordance with the Good Regulator principle.

At the earliest stage, these regulators operate independently. The corresponding self-representation Ω^0^ is therefore the set of independent models associated with these regulators:


$$R=\{R_{0},R_{1},\ldots R_{n}\}$$

$$\Omega =\{\;\rho_{0},\rho_{1},\ldots \rho_{n}\}$$

In this regime, there is no integrated internal space and no coordination between regulatory processes. Behavior is determined by local interactions between individual regulators and the environment.

Nevertheless, the system already exhibits a defining property of living systems: the maintenance of homeostasis through regulation. Importantly, this does not imply any form of intention or subjective striving. Apparent self-preservation arises as a consequence of structural stability: systems with effective regulation persist longer than those without it.

#### 3.2.3 Transition to coordinated regulation

While systems at the level Ω^0^ are capable of maintaining homeostasis through independent regulators, such an organization imposes strong limitations. Each regulator responds only to its own variable, without taking into account the global state of the system. As a result, different regulatory processes may interfere with each other or produce suboptimal outcomes when environmental conditions become more complex.

This limitation becomes particularly significant as the number of regulated variables increases. In a simple environment, independent regulators may be sufficient. However, in a changing environment where multiple factors simultaneously influence the system, maintaining homeostasis requires coordinated responses.

From an evolutionary perspective, this creates pressure toward the emergence of links between previously independent regulators. Over time, interactions between regulators become structured, such that the state of one regulator influences the activity of others. In this way, higher-order regulatory loops arise that coordinate multiple subsystems.

This transition marks the emergence of Ω^1^: a self-representation in which the models of regulators are no longer fully independent, but become functionally interconnected.

### 3.3 Coordinated self-representation and Ω^1^

At the level Ω^1^, regulatory models are no longer independent. Instead, they become interconnected, forming a system in which the state of one model can influence others. Formally, this corresponds to the appearance of functional dependencies between models:


$$\rho_{i}=f(\;\rho_{j},\rho_{k},\ldots )$$

These dependencies give rise to higher-order regulators that operate over multiple subsystems. As a result, the system is able to produce integrated responses based on the combined state of several variables.

This coordination significantly increases the robustness of homeostasis. Rather than maintaining each variable separately, the system can now regulate patterns of states across subsystems. This allows it to function effectively in more complex and variable environments.

This coordinated organization constitutes the first genuinely structured form of self-representation. Unlike *Ω*^0^, where Ω is simply a set of independent models, at Ω^1^ it acquires internal organization through the relations among regulators.

#### 3.3.1 Ω^1^ in the cell

The first emergence of Ω^1^ occurs already at the level of unicellular organisms. Cellular processes are not independent; they are coordinated through biochemical and enzymatic cascades, in which reactions regulate one another and form higher-order control structures.

In such systems, regulation is already integrated, but it remains entirely internal and distributed across molecular interactions. Although this coordination can be extremely complex, it does not yet provide a unified space for processing signals across different parts of a larger organism.

### 3.4 Emergence of the nervous system

#### 3.4.1 Ω^1^ in evolution

A qualitatively new stage in the development of Ω^1^ occurs with the emergence of *multicellular organisms* and the first *nervous systems*, approximately 600 million years ago, during the late Precambrian and early Cambrian periods.

The key innovation at this stage is the appearance of specialized cells whose primary function is signal transmission and coordination. This leads to the formation of *diffuse neural networks*, in which signals from different parts of the organism are collected and propagated within a shared structure.

For the first time, this creates a *unified regulatory space* at the level of the whole organism. Signals are no longer confined to local interactions within cells or tissues; they become part of a common network that integrates information across the organism.

This is a fundamental evolutionary transition. It establishes the physical basis for large-scale coordination and enables the organism to respond as a whole rather than as a collection of independent parts.

#### 3.4.2 Neurons as uniform processing units

From the perspective of TEVSER, the specific biological implementation of neurons is not essential at this stage. What matters is their functional role within the system.

In the simplest approximation, a *neuron* can be viewed as a unit that sums incoming signals and generates an output signal if a certain threshold is reached. In this sense, neurons do not “interpret” signals in a semantic way; they simply integrate inputs and propagate activity further through the network.

Crucially, in such a system, all neurons are functionally equivalent at the level of signal processing. Signals transmitted through the network have the same basic form, and their meaning is not encoded in the signal itself, but only in the structure of connections.

This has an important consequence. When signals from different regulators enter a common neural network, they are represented in a *uniform format*. The network can integrate and propagate them, but it does not inherently distinguish between signals of different origin or significance.

Thus, the same mechanism that creates a unified regulatory space also produces a fundamental limitation: signals become integrated, but not yet differentiated.

#### 3.4.3 Innate reflexes

At this level, behavior is primarily organized in the form of *innate (unconditioned) reflexes*.

An innate reflex is a fixed, evolutionarily established response linking sensory input to motor output through a predefined pathway. Such responses do not require learning and are present from the outset of the organism’s life.

Functionally, innate reflexes correspond to stable mappings within Ω^1^, where certain configurations of regulator states reliably trigger specific actions. These mappings are encoded in the structure of the system itself and are shaped by evolutionary selection. Formally, this corresponds to stable dependencies between models:


$$\rho_{i}\;(t)=f(\rho_{j}\;(t-1))\;\to a_{i}$$

Innate reflexes provide a simple but effective mechanism for interacting with the environment. They allow the organism to respond rapidly to relevant stimuli without the need for internal modeling of alternatives or past experience.

At the same time, they impose strong limitations. Behavior is rigid, context-independent, and determined entirely by current input and fixed internal connections. There is no mechanism for forming new associations or adapting responses based on experience.

This distinction is important, because later forms of learning (e.g., conditioned reflexes) emerge only on the basis of these pre-existing innate structures.

### 3.5 Emergence of differentiated signals and Ω^2^

#### 3.5.1 Need for differentiation

The emergence of a unified neural space at the level Ω^1^ significantly increases the integrative capacity of the organism. However, it also introduces a fundamental limitation.

Because neurons operate as uniform processing units—summing inputs and propagating signals in a common format—signals from different regulators become indistinguishable within the network. While the system can integrate and propagate activity, it lacks an intrinsic mechanism to differentiate signals according to their origin or functional significance.

This creates a critical problem. Effective regulation in a complex environment requires not only integration of signals, but also their differentiation. The system must be able to distinguish between signals corresponding to different regulatory processes and assign them different priorities. Without such differentiation, coordinated regulation cannot scale to more complex forms of behavior.

From an evolutionary perspective, this limitation becomes increasingly important as the number of regulatory variables and possible actions grows. Systems that develop mechanisms for distinguishing signals gain a significant advantage, as they can organize internal states more effectively and respond more selectively to environmental conditions.

#### 3.5.2 Structural differentiation and the labeled line principle

The most natural solution to the problem of signal differentiation is structural separation. Rather than encoding differences in the signals themselves, the system encodes them in its own organization: signals from different regulators become associated with distinct regions or pathways within the network.

Let us denote the distinguishing label of model ρ_i_ as ψ_i_. Then the full representation of regulator R_i_ model becomes:


$$R_{i}\to \psi_{i}\times \rho_{i}(S_{i})$$

$$\Omega^{2}⊃\psi_{i}\times \rho_{i}$$

In neuroscience, a closely related idea is known as the *labeled line principle*, according to which signals are distinguished by the specific pathways or regions through which they are transmitted. A well-known example is the *somatotopic organization of the cortex*, often illustrated by the Penfield homunculus (Penfield and Boldrey, 1937), where different parts of the body are mapped onto distinct cortical regions.

**Figure 1:**
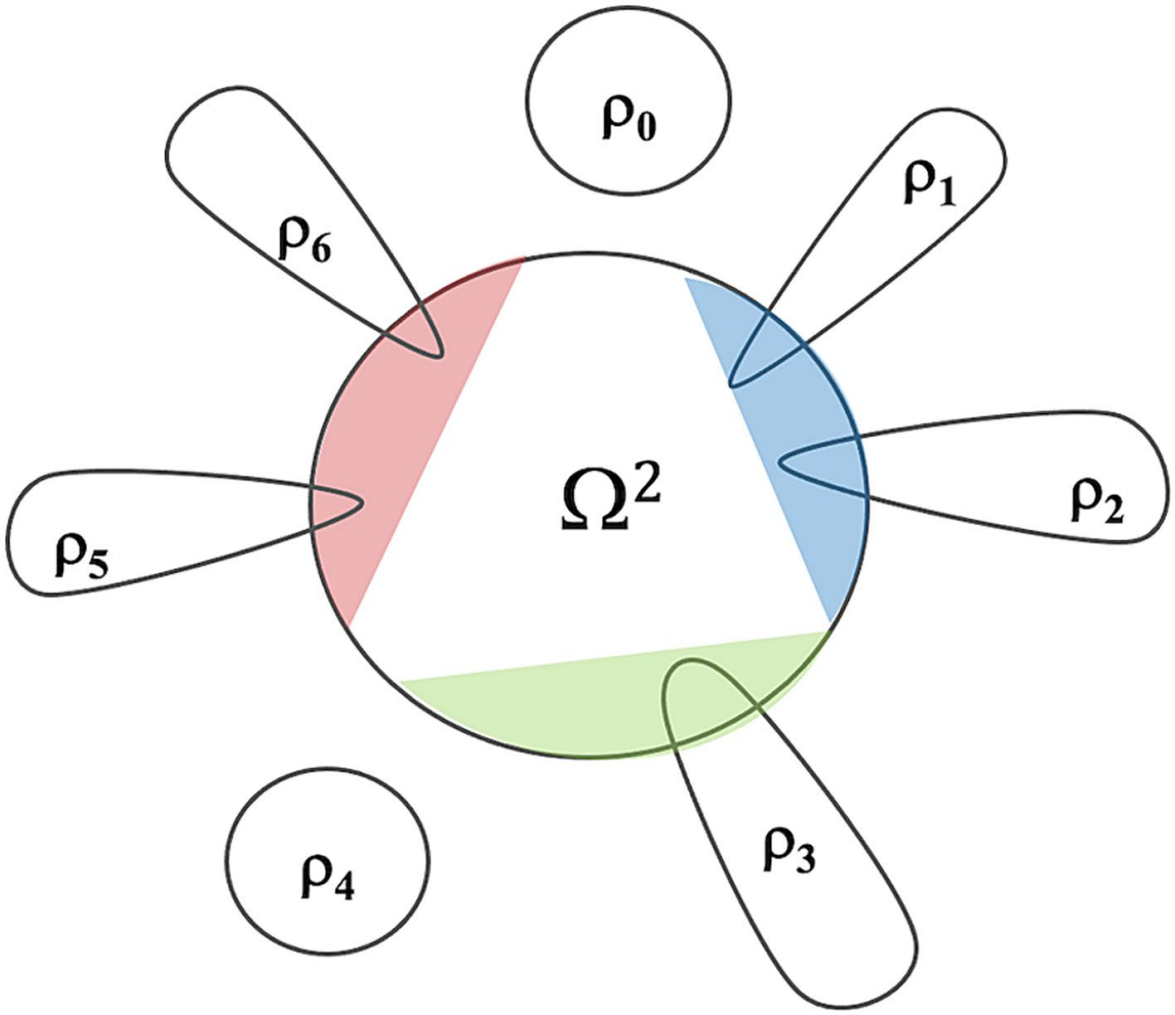
In self-representation Ω^2^ identity of a signal is determined not by its intrinsic form, but by its spatial location.

In such systems, the identity of a signal is determined not by its intrinsic form, but by its location within the network (Figure 1).

We propose that a similar principle operates at earlier stages of evolution. In the context of TEVSER, this suggests that the differentiation of signals at the level Ω^2^ arises through *spatial organization* of the system itself.

Notably, modern computers use a similar mechanism. When we write an expression in Python like Z = a*x + b*y, the compiler ultimately translates variables a, b, x and y into physical memory addresses. In other words, the system distinguishes them *by location* – just as biological systems did hundreds of millions of years ago.

#### 3.5.3 Ganglia as a physical realization

We hypothesize that a natural physical realization of this principle is the emergence of *ganglia*—spatially localized clusters of neurons associated with specific types of signals.

Importantly, within TEVSER this is not taken as a purely descriptive anatomical fact, but as a functional hypothesis: ganglia can be understood as structures that enable the differentiation of signals within a unified neural space by associating different regulatory processes with distinct regions.

In this sense, the spatial organization of ganglia provides a mechanism for labeling signals, transforming the weakly differentiated unified space of Ω^1^ into the structured internal domain of Ω^2^.

#### 3.5.4 Sensations

Within Ω^2^, internal signals corresponding to the states of individual regulators become *sensations*.

A sensation can be defined as the mapping of a regulator state ψ_i_ × ρ_i_ into the structured internal space of Ω^2^, preserving information about its origin. For example, signals related to temperature, mechanical damage, or chemical gradients are represented as distinct types of internal states.

Why does a sensation *feel* the way it does? Why does cold feel like *this*? This question can be approached constructively. Within TEVSER, the qualitative differences between sensations are not reduced to their functions – this point is developed further in the section on Psyche below.

#### 3.5.5 Emotions

In addition to localized signals, Ω^2^ includes global integrative states that reflect the combined influence of multiple regulators. These states modulate large parts of the system and influence behavior over extended periods.

In TEVSER, such states correspond to *emotions*.

Unlike sensations, which are tied to specific subsystems, emotions are distributed and act as a form of global coordination. They integrate signals from different sources into a unified value that influences action selection. In this sense, emotions function as a “common currency” of regulation.

From a neurophysiological perspective, such global states are consistent with the action of neuromodulatory systems, which affect large populations of neurons simultaneously.

#### 3.5.6 Psyche as a phenomenological domain

The combination of differentiated sensations and global emotional states constitutes the *psyche* of the system.

Within TEVSER, the psyche is defined as the complete set of sensations and emotions arising in the self-representation Ω^2^. Importantly, this definition is constructive: it is based on the functional role of these states in regulation, rather than on introspective description.

At the same time, we propose that this structured internal domain corresponds to what is traditionally referred to as a *phenomenological inner world*. In other words, once signals are differentiated and integrated within Ω^2^, the system does not merely process information—it possesses an internal domain of states that are experienced differently and influence behavior in distinct ways.

Differentiated regulatory states do not generate phenomenology as a downstream product; they *are* what the difference between signals amounts to, as seen *from within* the system. Phenomenology is not the outcome of function but its *inner side* – what function is for the system *itself*, not for an observer describing it from outside.

These differentiated states constitute *proto-qualia*: minimal forms of differentiated inner experience that arise with the first integrated regulatory space. Their full temporal depth and contextual richness develop at higher levels of self-representation.

Within TEVSER, the emergence of Ω^2^ marks the emergence of a primitive phenomenological world – the first minimal domain in which signals are not merely processed, but felt from within.

We acknowledge that this claim faces a fundamental methodological challenge: phenomenal experience cannot be directly observed from a third-person perspective (Nagel, 1974). The most viable empirical strategy is to identify neural signatures of differentiated inner states in humans, and to use these as a basis for comparative inference in other species – a strategy already employed in consciousness research more broadly.

The hypothesis about Ω^2^ can be illustrated by simple organisms. For example, in the nematode *C. elegans*, specific neurons respond to changes in environmental conditions (such as temperature), while others activate under critical thresholds (e.g., a “too hot” neuron), triggering global modulatory responses. These responses influence behavior in a coordinated way and persist over time.

In the language of modern biology, such states are often described as “behavioral states.” However, within the TEVSER framework, they can be interpreted as a minimal form of sensations and emotions, and therefore as a primitive psyche.

This does not imply that such organisms possess consciousness or reflective awareness. Rather, it suggests that the foundations of a phenomenological domain emerge earlier in evolution, as soon as the system acquires differentiated internal states within a unified representation.

This includes nociceptive states: within TEVSER, an organism with Ω^2^ that responds to noxious stimuli does so not merely as a reflex, but as a system with a differentiated internal domain in which aversive signals are *felt differently* from neutral ones.

On the ontological commitments of TEVSER, this constitutes a minimal form of pain – not the rich, conscious suffering associated with human experience, but a genuine aversive inner state. The difference between the nociceptive experience of a nematode and the pain of a human is, within this framework, a difference of degree along the hierarchy of self-representation, not a difference in kind.

#### 3.5.7 Ω^2^ in evolution

From an evolutionary standpoint, Ω^2^ likely emerges in early multicellular organisms with increasingly centralized or semi-centralized nervous systems, approximately 570 million years ago, during the Ediacaran period, possibly giving rise to *Avalon explosion*.

At this stage, diffuse neural networks begin to give way to more structured organizations, including ganglia, where signals from different subsystems are integrated and differentiated within spatially organized regions.

Such structures provide the physical basis for the differentiation of signals and the emergence of a primitive psyche. This level is likely present in a wide range of early animals possessing nervous systems capable of integrating multiple types of input.

### 3.6 Self-localization and Ω^3^

#### 3.6.1 From psyche to subjectivity

At the level Ω^2^, the organism possesses a differentiated internal domain of sensations and emotions—a primitive psyche. However, the presence of such a domain does not yet imply subjectivity.

The distinction between psyche and subject is subtle and cannot be directly established from introspection alone. From a constructive perspective, it is useful to treat subjectivity as a property that emerges gradually in evolution.

In TEVSER, we distinguish several levels of the subject:

- the “subject here,”
- the “subject now” (introduced later in connection with consciousness),
- the “subject who” (associated with self-consciousness).

The present section focuses on the earliest form—the subject “here.”

#### 3.6.2 Subject “here”

The primary subject emerges as a feeling of one’s own existence as separate from the world. This is not reflection, but a direct, non-conceptual form of experience.

An organism at this level does not represent itself explicitly and does not reflect on its own existence. However, it experiences the world from a first-person perspective. The environment is perceived as a space containing food, threats, and other relevant elements, while the organism itself is implicitly present within this space.

Such agents do not yet have explicit goals. Instead, their behavior is driven by regulatory states (e.g., hunger), which give rise to directed activity such as searching for food.

#### 3.6.3 Localization of the self: the role of stereo sensations

We propose the hypothesis that subjectivity emerges with the appearance of mechanisms that allow an organism to *localize itself in space*.

The key requirement for such localization is the presence of *stereo sensations*—that is, pairs of spatially separated sensors (e.g., eyes, ears, olfactory structures) whose signals are processed jointly.

A single sensor provides only local information. In contrast, multiple spatially separated sensors allow the system to infer relative position by comparing signals. This comparison makes it possible to determine not only the presence of a stimulus, but its location relative to the organism.

For this to occur, the signals from these sensors must be projected into the psyche (Ω^2^) and processed within a unified internal space. The combination of:

- a unified representational domain (from Ω^2^), and
- stereo sensory input

Creates the conditions necessary for spatial self-localization.

#### 3.6.4 Ω^3^: spatial self-representation

At the level Ω^3^, the self-representation Ω includes not only internal states, but also a representation of the organism’s position relative to the surrounding environment.

Formally, this can be understood as the emergence of a coordinate system in which the organism occupies a central position (x_0_,y_0_) and the surrounding space is organized relative to this point.

As a result, Ω^3^ contains:

- a representation of the organism itself,
- and a representation of a local region of the environment *ε*(x_0_,y_0_)

**Figure 2:**
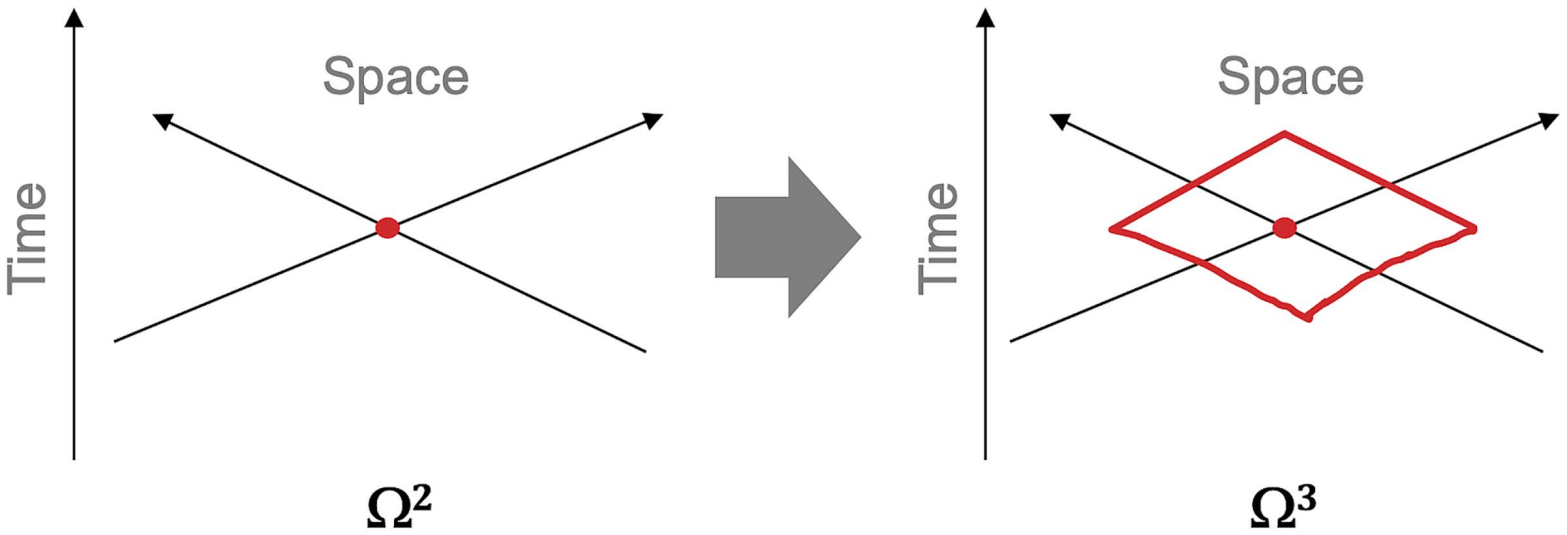
Evolution of self-representation from Ω^2^ (a model of the internal space) to Ω^3^ (a model of the surrounding space and of oneself within that space). The Z-axis represents time, while the X and Y axes represent space.

We will illustrate this idea of spatial self-localization using the model of a Minkowski light cone (Figure 2).

This represents a fundamental extension of the concept of self-representation. While earlier levels involve only models of internal regulatory processes, Ω^3^ includes models of the external world insofar as they are relevant to maintaining homeostasis.

At first glance, this may seem to contradict the original definition of self-representation as the complete set of internal models of all system regulators. But this extension is necessary because external events—such as the approach of a predator—can directly disrupt the internal stability of the organism. Therefore, from the perspective of long-term survival, regulation must include not only internal variables, but also relevant aspects of the environment.

#### 3.6.5 Ω^3^ in evolution

The emergence of Ω^3^ is likely associated with the appearance of *bilaterian organisms*, approximately 550 million years ago, during the early Cambrian period.

Bilateria are characterized by:

- bilateral symmetry,
- paired sensory organs,
- and increasingly structured nervous systems, often including ganglia.

In contrast:

- earlier organisms either lack a nervous system,
- or possess only diffuse neural networks without clear spatial differentiation.

The appearance of paired sensory structures enables stereo perception, and thus spatial localization. This marks a major evolutionary transition.

It is reasonable to hypothesize that the emergence of Ω^3^ contributed to the *Cambrian explosion* (~540–510 million years ago), during which most major animal body plans appeared.

This period is also associated with the emergence of the *predator–prey* interaction model, which in fact requires the ability to localize both oneself and other organisms in space.

#### 3.6.6 Examples and minimal cases

Within TEVSER, among modern organisms, some of the simplest agents that may instantiate the “subject here” are nematodes.

For example, in *C. elegans*, paired sensory structures such as amphids allow the organism to detect chemical gradients and localize itself relative to sources of stimuli (e.g., food). This enables spatially organized behavior despite the simplicity of the nervous system.

### 3.7 Learning and Ω^4^

#### 3.7.1 From self-localization to elementary learning

The capacity for elementary learning does not require spatial self-localization as a biological prerequisite – conditioned reflexes are observed in organisms that likely lack a fully integrated spatial perspective. Within TEVSER, Ω^4^ follows Ω^3^ in the hierarchy because both represent progressive expansions of the system’s regulatory reach: one into space, the other into time.

Nevertheless, even an organism that can distinguish itself from the surrounding world and orient in space remains limited if it can act only through fixed innate reflexes. As environments become more variable, a purely built-in repertoire of responses is no longer enough to maintain homeostasis over time.

Learning eventually becomes one of the key mechanisms of survival. In TEVSER, the first building block of learning appears already at this relatively early stage of evolution. At this point, the model of the world is still extremely primitive, but the organism already possesses a structured domain of sensations and actions. This makes it possible to form new connections between events occurring in close temporal proximity.

#### 3.7.2 Conditioned reflexes

The simplest form of learning is the association of two events, or more precisely, two consecutive states of sensors or actuators, that lead to a desirable or undesirable outcome.

In biology, this mechanism is well known as the *conditioned reflex*, first systematically described in the works of Sechenov (1863) and Pavlov (1903).

Conditioned reflexes are the simplest adaptive programs for interacting with the environment. At this stage, there is still no modeling of alternative possibilities, as will later appear in intelligence. Instead, there is a rigid linkage between two consecutive events at the level of sensations and actions.

Such systems are often described as *dynamic stereotypes*, emphasizing that behavior consists of stable, repeatedly executed sequences formed through experience.

The formation of conditioned reflexes is a highly effective mechanism, but it has important limitations:

1. Classical conditioning can operate across a range of inter-stimulus intervals, from subsecond to several seconds, depending on the paradigm and species. However, this form of association is inherently limited in its temporal reach: it works best over short intervals and cannot support learning of complex sequences or long-range causal relationships.
2. Stronger stimuli dominate weaker ones: if several signals precede the same outcome, the conditioned reflex is more likely to form around the stronger stimulus.
3. Familiar repeated stimuli are less likely to become associated with an event than novel or rare ones, a phenomenon known as latent inhibition.
4. Once a conditioned reflex is established, it becomes relatively stable, and new competing associations are less likely to form around the same event sequence.

These simple constraints make conditioned reflexes a remarkably effective early mechanism of learning in changing environments.

#### 3.7.3 Ω^4^ emergence in evolution

To understand when conditioned reflexes appeared, it is useful to compare them with innate reflexes.

Innate reflexes are stable, inborn responses to specific stimuli. They are implemented through fixed reflex arcs, in which neural signals travel from receptor to sensory pathway, through a processing center, and then through a motor pathway to an effector organ. Their structure is predetermined and does not require learning.

Conditioned reflexes differ from innate reflexes in three essential respects:

- First, the reflex arc is formed during the lifetime of the organism rather than being fully present from the start.
- Second, this acquired arc may weaken or disappear if the corresponding pairing is not reinforced.
- Third, a conditioned reflex can only arise on the basis of an already existing innate reflex.

In modern animals, conditioned reflexes are typically associated with forebrain structures and, in more complex organisms, with the cerebral cortex. However, experimental evidence suggests that conditioned reflexes already exist in simple bilaterians lacking a fully developed forebrain. For example, they have been demonstrated in nematodes such as *C. elegans*, whose nervous system contains only a circumpharyngeal nerve ring, structurally comparable to a single circular ganglion (Hale et al., 2016).

For this reason, it is reasonable to assume that conditioned reflexes began to emerge in early bilaterian organisms possessing at least a rudimentary forebrain or an equivalent integrative structure.

#### 3.7.4 Ω^4^: self-representation with acquired rules

Thus, self-representation level Ω^4^ includes not only models of individual sensors and actuators, and not only fixed linkages between their states, but also the ability to *form new rules* connecting events during the lifetime of the organism.

In TEVSER, this means that Ω^4^ introduces acquired transitions between elements of self-representation:


$$S⊃S_{i}$$

$$R_{i}\to \rho_{i}(S_{i})$$

$$\exists \rho_{j}:\rho_{j}\;(t+1)=f(\rho_{i}\;(t))$$

$$\Omega^{4}⊃\rho_{j}$$

The crucial point is that these are still short-range, rigid, experience-dependent linkages, but the system already does more than execute innate reflexes: it can acquire new behavioral couplings from experience.

This marks the emergence of the first genuinely adaptive form of learning within the hierarchy of self-representations.

### 3.8 Attention and Ω^5^

#### 3.8.1 Attention

At the previous stages, the agent already possesses a psyche (Ω^2^), a basic subject (“here,” Ω^3^), and simple learning mechanisms (Ω^4^). However, the system remains limited: it processes incoming signals more or less uniformly and lacks a mechanism for dynamically prioritizing them in real time.

At a certain point in evolution, a new function emerges—*attention*.

Attention is a mechanism that allows the system to selectively enhance some signals while suppressing others. In other words, not all incoming information is processed equally: some signals become dominant at a given moment, while others are attenuated.

This introduces a fundamentally new property: the system no longer treats all internal states symmetrically, but instead dynamically allocates processing resources.

As a result, behavior becomes significantly more efficient. Instead of reacting to all stimuli simultaneously, the agent can focus on the subset of signals most relevant for maintaining homeostasis at that moment.

#### 3.8.2 Ω^5^: self-representation with dynamic prioritization

The emergence of attention leads to a new level of self-representation—Ω^5^.

At this level, the system includes:

- differentiated signals (as in Ω^2^),
- spatial self-localization (as in Ω^3^),
- learned associations (as in Ω^4^),
- and now, dynamic prioritization of signals.

Thus, the system not only represents the world and itself, but also continuously selects which elements of this representation are currently dominant.

Formally:


$$S⊃S_{i}$$

$$R_{i}\to \rho_{i}(S_{i})$$

$$\forall t:\exists n:A_{n}>>A_{i},i!\neq n$$

$$\Omega^{5}\;(t)⊃A_{i}\times \psi_{i}\times \rho_{i}$$

At any given moment, one signal (or a small subset) dominates over others, forming a transient focus of processing. Here, Aᵢ denotes the attention weight assigned to signal ρ_i_, with the dominant signal defined as the one with the highest current weight.

This can be interpreted as a dynamic window of relevance, which shifts over time depending on the internal state of the organism and external conditions.

#### 3.8.3 Functional role of attention

Attention does not introduce new types of representations. Instead, it reorganizes access to existing ones.

Its primary function is to:

- reduce the effective dimensionality of processing,
- enable rapid decision-making in complex environments,
- and ensure that behavior is driven by the most relevant signals at each moment.

Importantly, attention operates continuously and automatically, without requiring explicit control or reflection.

#### 3.8.4 Ω^5^ in evolution

The emergence of Ω^5^ is likely associated with organisms possessing:

- well-developed sensory systems (especially vision),
- and mechanisms for actively controlling perception.

This stage is most plausibly reached in early *vertebrates*, approximately 500 million years ago, in the early Cambrian.

In vertebrates, a key biological implementation of attentional selection is the *control of eye movements*.

In vertebrates:

- the midbrain (optic tectum) plays a central role in visual processing,
- oculomotor systems allow the organism to orient toward specific stimuli,
- and gaze direction becomes a mechanism for selecting relevant parts of the environment.

This provides a concrete biological implementation of attentional selection. The oculomotor example is a well-studied biological implementation of attentional selection in vertebrates; other organisms may achieve similar selection through different mechanisms.

In higher organisms, additional cortical mechanisms allow voluntary control of attention, but the fundamental mechanism originates from these evolutionarily ancient structures.

### 3.9 Object differentiation, memory and Ω^6^

#### 3.9.1 Need to reduce complexity

Sensory systems produce a massive amount of raw information. For example, visual input alone may contain a large number of “pixels,” each carrying detailed information about color, intensity, and motion. Processing this information directly is computationally expensive and time-consuming.

At the same time, the environment becomes increasingly dynamic. Rapid decisions are required, often under conditions where delays can be fatal.

This creates a fundamental paradox: the richer the sensory input, the more resources are required to process it, yet the faster decisions must be made.

From an evolutionary perspective, this makes it inevitable that mechanisms arise to reduce the complexity of raw sensory input.

#### 3.9.2 Object differentiation

The natural solution is the extraction of *objects* from sensory data.

Instead of processing raw signals, the system identifies coherent entities—objects—and operates on them. This dramatically reduces complexity and allows faster decision-making.

In TEVSER, this corresponds to the emergence of mappings:


$$\exists \varphi :\;Z_{i}\to O_{i}$$

Where external objects Z_i_ are transformed into internal representations ρ_j_.

Thus, self-representation now includes internal models of external objects. Here, Z_i_ denotes an external object in the environment, and O_i_ denotes its internal representation within the system’s self-model.

Object differentiation can also be understood as an extension of attention. To distinguish an object, the system must direct attention to a region of space and effectively “probe” it to determine its boundaries.

#### 3.9.3 Memory

Memory is the ability to preserve and later use information about past states of the system and its environment.

From the TEVSER perspective, memory is an *extension of self-representation into time*, acquiring ability to store past states.

Within TEVSER, the form of memory emerging at this level is closely tied to object differentiation: rather than storing raw sensory input in its entirety, the system stores compressed representations – already identified objects and their states.

The recognition of the same object over time is itself a minimal form of memory, illustrating how object differentiation and this form of memory co-emerge. Other forms of memory – such as spatial or procedural memory – are not dependent on object-level representations and are associated with earlier or parallel levels of the hierarchy.

At this stage, the most important form of memory is short-term memory.

Objects move in space, and the organism itself moves as well. Taking recent states into account and adjusting behavior accordingly becomes essential for effective interaction with the environment

#### 3.9.4 Subject “now”

The emergence of memory leads to a new form of subjectivity—*the subject “now.”*

Just as localization in space revealed the *extent of space* and gave rise to the subject “here,” the emergence of memory reveals the *sequence of events* and gives rise to the subject “now.”

The subject now exists not only as a point in space, but as the endpoint of a trajectory. It has a past and a present, even if it does not yet possess an explicit concept of time.

This temporal extension of self-representation reveals the structure of events and enables the system to act based not only on the current state, but also on recent history.

Experimental observations support this idea. For example, in fish, it has been shown that if an aversive event follows a stimulus after a fixed delay (e.g., 5 s), the organism can learn to perform an action precisely at that delay to prevent the event (Portavella et al., 2004; Broglio et al., 2003).

This indicates the presence of a temporal structure in behavior—a hallmark of the subject “now.”

#### 3.9.5 Possible emergence in evolution

To understand the emergence of Ω^6^, it is useful to distinguish two functionally different types of memory.

##### 3.9.5.1 Memory of places

The first type is spatial memory, which supports navigation and orientation in the environment.

This form of memory is associated with structures homologous to the *medial cortex (hippocampus)*, which appear early in vertebrate evolution, approximately 500 million years ago.

It is important to note that the subject “here” (spatial self-localization) emerges earlier, in bilaterians (~540–550 million years ago). However, the ability to retain and use information about spatial locations—i.e., to remember places—develops later.

##### 3.9.5.2 Memory of objects and events

The second type is memory of objects and events.

Unlike spatial memory, this form of memory develops organically within the same neural structures responsible for object differentiation. The neural basis of this co-emergence in biological systems has been studied extensively in the perirhinal cortex (Bussey and Saksida, 2005; Brown and Aggleton, 2001).

This relationship can be observed even in artificial systems: in modern neural networks, object recognition and the representation of those objects (i.e., their “memory”) are formed within the same architecture (LeCun et al., 2015).

This component of self-representation likely began to develop around 370 million years ago, particularly in early *amphibians* (Deryagina, 2003).

At this stage:

- the forebrain becomes more developed,
- a primitive cortical structure appears—the *archipallium* (ancient cortex),
- and connections between forebrain and midbrain are strengthened.

The archipallium is a relatively simple, three-layered structure, yet it already performs key functions of object differentiation. In this sense, it can be regarded as an early biological analogue of a convolutional network, extracting structured representations from sensory input (Northcutt and Kaas, 1995; Striedter, 2005). Unlike artificial networks, biological memory involves a distributed architecture in which encoding, consolidation, and retrieval are supported by partially distinct structures and processes. The analogy here concerns computational function, not implementation.

#### 3.9.6 Gradual increase in complexity

At early stages, object differentiation and memory are likely to have been coarse and imprecise. The internal representations of objects would have been vague and unstable compared to those of modern animals.

However, evolutionary processes favor incremental improvements. Over time:

- object representations become more stable,
- memory becomes more reliable,
- and the number of distinguishable objects increases.

This gradual refinement leads to the situation observed in modern organisms, including humans, where a large number of objects can be simultaneously recognized, differentiated, and recalled.

#### 3.9.7 Ω^6^: self-representation extended in space and time

At the level Ω^6^, self-representation includes:

- internal models of external objects,
- and representations of past states.

Formally:


$$Z⊃Z_{i},\;Z_{i}\cup S=\emptyset$$

$$\exists \varphi :\;Z_{i}\to O_{i}$$

$$\Omega^{6}⊃O_{i}$$

$$\exists \rho_{k}:\rho_{k}\;(t)⊃\rho_{k}\;(t-1),\rho_{k}\;(t-2),\ldots$$

Thus, self-representation is extended not only into the surrounding region of space, but also into the recent past.

**Figure 3:**
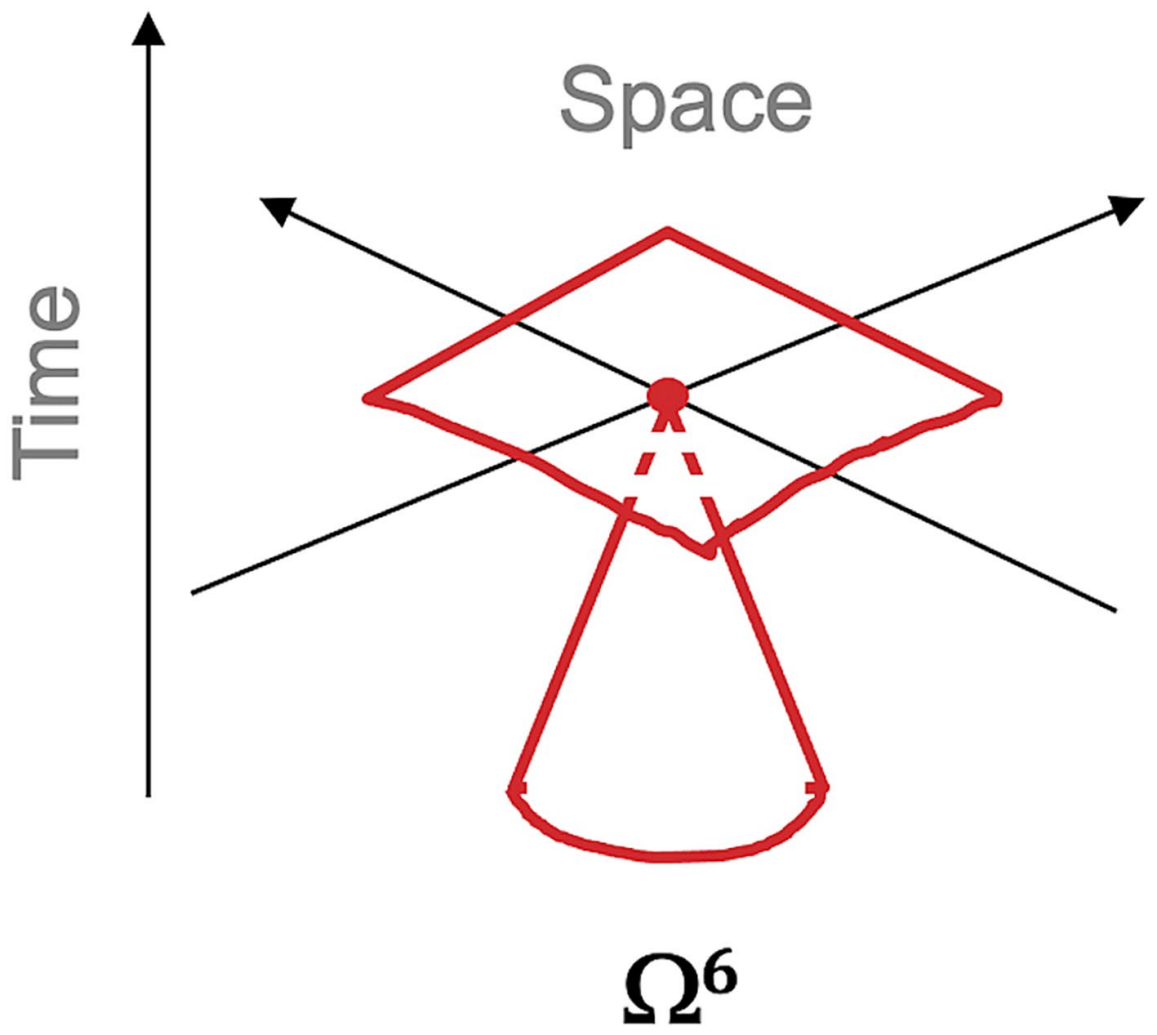
Self-representation Ω^6^ includes not only the nearby region of space, but also the nearby region of past events. The Z-axis represents time, while the X and Y axes represent space.

In this sense, Ω^6^ can be interpreted as including a fragment of the past light cone of events within the system’s internal representation (Figure 3). We use the light cone here as a structural analogy: just as the past light cone of an event contains all points in space–time that could have causally influenced it, self-representation at this level encompasses the nearby region of space and the recent past. This is a structural parallel, not a claim about relativistic physics.

### 3.10 Consciousness

At this point, all necessary components for conscious experience are in place and we can formulate a constructive definition of consciousness.

Consciousness arises as the combination of:

- the psyche (Ω^2^),
- attention (Ω^5^),
- the subject “here” (Ω^3^),
- and the subject “now” (Ω^6^).

Thus, within the TEVSER framework:


$$\text{Consciousness}=\text{psyche}+\text{attention}+{\text{subject}}^{“}{\text{here}}^{”}+{\text{subject}}^{“}{\mathrm{now}}^{”}$$

This definition should be understood as a minimal constructive condition rather than a sufficient characterization of all forms of consciousness.

This definition should not be read as claiming that consciousness is absent below Ω^6^ and suddenly present at it. Each component in this definition represents a form of experience already present at earlier levels: the psyche introduces differentiated inner states at Ω^2^, spatial perspective appears at Ω^3^, attentional modulation at Ω^5^.

What Ω^6^ marks is the first level at which all these dimensions are simultaneously integrated—producing the form of conscious experience that humans typically recognize as such in everyday life. Consciousness, in TEVSER, is not a threshold but a progression.

At this stage, the organism possesses a full internal phenomenal world structured in both space and time. The organism experiences the world from a first-person perspective, with attention dynamically selecting elements of the environment. Different objects enter and leave the focus of attention, forming a continuous stream of experience.

However, it still lacks *self-consciousness*—the ability to explicitly represent and reflect on itself as an object. A useful analogy is the *flow state* described by Csikszentmihalyi (1975). During such states, an agent is fully immersed in interaction with the environment, with highly focused attention and efficient behavior, but without explicit self-reflection.

### 3.11 Qualia

At Ω^6^, the system acquires full qualia: qualitatively differentiated internal states embedded in temporal and spatial context.

The emergence of qualia at this level does not mark the first appearance of subjective experience in the TEVSER hierarchy. Proto-qualia – minimal forms of differentiated inner experience – arise already at Ω^2^, where different regulatory signals begin to be felt differently from within the system. What Ω^6^ introduces is their full temporal and contextual depth: qualia here are not merely differentiated signals, but situationally embedded, memory-connected, spatially and temporally structured experience.

In this sense, TEVSER describes not a single threshold of subjective consciousness, but a progression of increasingly rich forms – from proto-qualia (Ω^2^), through the emergence of a spatial perspective and the subject “here” (Ω^3^), through attentionally modulated experience (Ω^5^), to the full phenomenological world (Ω^6^).

Within TEVSER, qualia are therefore not intrinsic properties of external stimuli, nor separate non-physical entities. They are how the system’s internal states are organized and experienced *from within* – at the level where self-representation has become temporally extended, spatially anchored, and memory-integrated. A detailed account of their structure and functional role is given in Section 5.3.

### 3.12 Behavioral intelligence

#### 3.12.1 The subject “future”

Intelligence, understood as the ability to predict the future, is a natural continuation of the evolution of self-representation in the context of maintaining homeostasis in space–time.

At earlier stages:

- Ω^1^ represents internal spatial domain,
- Ω^3^ introduces a representation of local environment around,
- Ω^6^ extends representation into the past.

However, as environments become more dynamic, the number of possible future developments increases. Each of these may either support or threaten homeostasis.

This creates evolutionary pressure for the emergence of self-representations that include possible future states.

Thus, intelligence can be defined as:

the ability to model possible future behaviors and outcomes, and to select among them.

**Figure 4:**
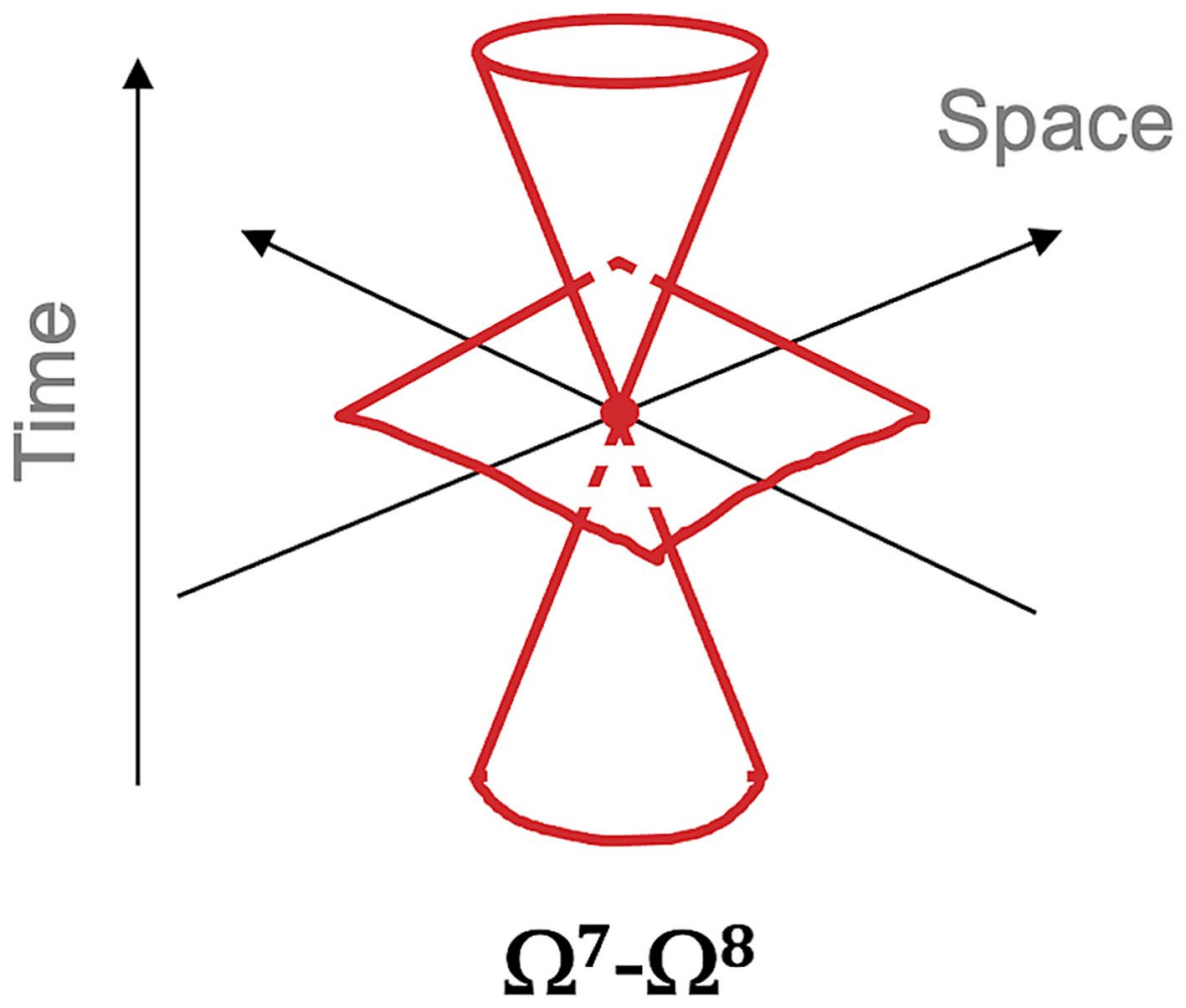
Intelligence is an extension of self-representation Ω into the future. The Z-axis represents time, while the X and Y axes represent space.

In TEVSER terms, this corresponds to extending self-representation into the future light cone of events, complementing its existing extension into space and past time (Figure 4).

Behavioral intelligence includes:

- the development of reinforcement learning (Ω^7^),
- and the subsequent emergence of hierarchical predictive intelligence (Ω^8^).

#### 3.12.2 Causal relationships

Prediction of the future relies on detecting regularities in the world.

Events do not occur randomly: some consistently follow others. If event A frequently precedes event B, then the occurrence of A increases the likelihood of B.

This forms a *cause-and-effect relationship* (CER).

Such relationships can involve:

- purely external events,
- purely internal states,
- or combinations of both.

Intelligence consists in:

- detecting these relationships,
- constructing them within self-representation,
- and using them to predict future events.

Importantly, most predictions remain unconscious. Only prediction failures bring processes into awareness—for example, when a step is missing while walking.

At higher levels, intelligence actively searches for CER, which likely explains the human attraction to narratives—structured sequences of cause and effect.

However, CER formation is biased, the brain tends to form associations more easily than to discard them, which explains the persistence of incorrect beliefs.

#### 3.12.3 Neural connections as CER representation

From modern neuroscience and artificial neural networks, we know that events and objects correspond to patterns of neural activation.

If two events are represented by two neural populations, then a causal relationship between them corresponds to a connection between these populations. And if one event follows another within a short time interval, then a connection between them becomes stronger.

This is consistent with Hebb’s rule (Hebb, 1949):

“cells that fire together wire together.”

Thus, TEVSER proposes:

Neural connectivity evolved as a mechanism for encoding causal relationships.

At this level of the hierarchy, temporal co-occurrence serves as a primitive form of causal representation: the system treats ‘A preceded B’ as a functional equivalent of ‘A causes B.’ This is the foundational mechanism, first present at Ω^4^.

At Ω^7^, causal encoding becomes more sophisticated – a distributed, state-dependent process involving broader network dynamics, prior knowledge, and internal context – enabling the system to evaluate not just individual associations, but entire sequences of states and their expected consequences.

### 3.13 Reinforcement learning and Ω^7^

#### 3.13.1 Trial and error learning

As causal chains grow longer, not all of them are equally relevant. From a survival perspective, the system must distinguish:

- beneficial outcomes (to be pursued),
- harmful outcomes (to be avoided).

Thus, causal relationships must acquire *value*. The easiest way to obtain this value is by direct experience – through trial and error.

This leads to *reinforcement learning*, where:

- behavior is evaluated through experience,
- positive outcomes reinforce actions,
- negative outcomes suppress them.

This principle was discovered and formalized in Thorndike (1898) *law of effect*.

Biological principles of this type of learning (specifically, temporal-difference learning), were described by Sutton (1978) and Schultz et al. (1997):

- the hypothalamus encodes internal state,
- dopamine signals expected reward,
- the basal ganglia select actions.

While in bilaterians dopamine was a signal of reward, in vertebrates, dopamine reflects *reward prediction*, not reward itself:

- increased expectation → increased dopamine,
- violated expectation → sharp decrease.

This mismatch between predicted and received reward is known as the *reward prediction error* – the key teaching signal of temporal-difference learning (Schultz et al., 1997). A related signal is *salience*: the degree to which a stimulus stands out and demands attentional resources, regardless of its immediate value. Both reward prediction error and salience contribute to directing the system’s behavior toward biologically significant events.

#### 3.13.2 Self-representation level Ω^7^

At this level, self-representation includes:

- possible states ω_i_,
- possible actions α_j_,
- and a value of states Q(ω_i_).

Thus, the system selects actions not only to maintain homeostasis, but to maximize expected value:


$$\Omega^{7}⊃Q\;(\omega_{i})$$

αt=argmaxQ(α)

This represents a fundamental shift:

- from reactive behavior → to value-based decision-making.

#### 3.13.3 Ω^7^ in evolution

Within TEVSER, the transition at this level does not mark the first appearance of reward-based learning, but the emergence of an explicit state-value representation associated with the differentiation of the *basal ganglia (striatum)* in early vertebrates. Damage of striatum impairs this form of learning, confirming its central role in state-value computation (Cognigni et al., 2018).

Earliest significant enlargement of striatal bodies was shown in *early reptiles* (~310 million years ago). Also, for the first time, reptiles show the emergence of a rudimentary neocortex (neopallium) (Deryagina, 2003).

This level of adaptation may have contributed to the long evolutionary dominance of reptiles (dinosaurs).

### 3.14 Hierarchical intelligence and Ω^8^

#### 3.14.1 Brain as the predictive system

The ability to learn from direct experience during lifetime allowed organisms to build simple predictions about future situations and to quickly adapt to their environment. However, reinforcement learning still requires direct experience, which is risky.

But the true power of prediction is the ability to *simulate future scenarios internally*.

The idea of the brain as a predictive system has deep historical roots, beginning with Hermann von Helmholtz, who introduced the concept of *unconscious inference*, framing perception as an active process of hypothesis generation about the causes of sensory input.

This intuition was later given a concrete computational and neural formulation by Rao and Ballard (1999), who proposed hierarchical predictive coding, in which higher cortical areas generate top-down predictions while lower areas propagate bottom-up prediction errors.

In parallel, similar ideas were independently developed and popularized by Hawkins and Blakeslee (2004) and Hawkins and Subutai (2016), particularly in the context of sequence learning and cortical organization, describing the neocortex as a hierarchical system for learning and predicting temporal patterns.

These approaches were subsequently unified and formalized within the framework of active inference by Friston (2005, 2009, 2010) and Friston et al. (2006), who cast the brain as a hierarchical generative model minimizing variational free energy, thereby integrating perception, action, and learning into a single inferential process.

*Key principles*:

- top-down predictions
- bottom-up prediction errors
- hierarchical organization of representations

*Hierarchy*:

- Higher layers: encode abstract, long-term patterns
- Lower layers: process immediate sensory input

Higher levels continuously build models of future and predict future states, while lower levels continuously report actual outcomes. The mismatch between prediction and reality (prediction error) drives both learning and adjustment.

#### 3.14.2 Goals and motivation

From the perspective of active inference, this level introduces a fundamentally new form of behavioral control in the agent—the ability to *set goals*.

At the level of hierarchical intelligence (Ω^8^), higher layers generate possible future scenarios and, having selected an optimal one, propagate a corresponding plan of actions downward to lower sensorimotor levels. The agent then begins to act in order to realize this plan.

However, a critical question arises: what happens when reality diverges from the prediction?

- prediction: *“I should observe X”*
- reality: *“I observe Y”*

This mismatch gives rise to a prediction error, which the system can reduce in two principal ways:

- by updating its internal model (perception): *“the world is not X, but Y,”*
- or by acting on the world (action): *“I will act so that it becomes X.”*

Thus, goal-directed behavior emerges as the attempt of the agent to *change the world to match its selected model of reality*.

Importantly, hierarchical intelligence does not merely select the next action from a set {a_0,_…a_K_}. Instead, it addresses a more general question:

*“If I already expect to observe X, which sequence of actions will lead to X?”*

In this sense, behavior becomes a form of *self-fulfilling inference*, where the system selects actions in order to obtain “convenient” observations consistent with its predictions.

Accordingly, goal striving can be understood as a special case of minimizing the discrepancy between predicted and actual sensory input. Goals emerge in situations where updating the internal model becomes less preferable than changing the external world.

Within this framework, *motivation* arises as the gradient of prediction error reduction: the greater the discrepancy, the stronger the drive to minimize it. What is externally interpreted as the system’s “desire” to achieve a goal is, in fact, a manifestation of the dynamics of minimizing mismatch with its own prior expectations.

#### 3.14.3 Goals as virtual regulators

From the perspective of TEVSER, goal setting and plan generation represent a special case of a more general mechanism—the formation of regulators.

At the level Ω^8^, the system constructs a *virtual regulator* in the space of self-representation:

- the goal serves as the model (desired state),
- the plan serves as the regulatory mechanism driving the system toward that state.

Thus, self-representation acquires the ability to create second-order regulators, not tied directly to immediate homeostatic variables.

As a result, the system can:

- construct new preferred distributions,
- evaluate their attainability,
- and initiate the corresponding inference processes (actions).

#### 3.14.4 From regulation to behavioral intelligence

This expansion represents a fundamental transition.

At earlier levels, regulation is reactive and tied to predefined variables.

At Ω^8^, the system becomes capable of proactively shaping its own space of admissible states and trajectories.

This is precisely what defines *behavioral intelligence*: the ability not only to respond to deviations, but to construct, evaluate, and realize desired future states.

This interpretation allows a direct comparison with established physiological theories of goal-directed behavior.

#### 3.14.5 Agreement with the theory of functional systems (TFS)

The formulation of a goal as a “virtual regulator” reveals clear parallels with the TFS, proposed by Anokhin (1973).

A central concept of TFS is the *acceptor of the action result*—an internal model of the expected outcome against which the actual result of behavior is compared. Functionally, this acceptor plays the role of a goal: it specifies the expected state of the system and provides a criterion for evaluating the success of an action.

In this sense, the notion of a virtual regulator in TEVSER can be interpreted as a generalization of the acceptor of the action result, elevated from the level of specific physiological mechanisms to the level of self-representation.

#### 3.14.6 Relation to active inference

Within the framework of active inference, developed by Karl Friston, similar ideas receive a formal mathematical interpretation.

In this context:

- the acceptor of the result can be viewed as a prior distribution over expected sensory states,
- while the comparison between expected and actual outcomes corresponds to prediction error minimization.

From this perspective, active inference can be understood as a Bayesian formalization of the principles underlying TFS, embedding them within a probabilistic framework of perception and action.

#### 3.14.7 Extension in TEVSER

However, TEVSER extends this concept in an important way.

In TFS, the acceptor of the action result is typically formed in the context of solving a specific adaptive task, closely tied to the organism’s current physiological needs.

In contrast, within TEVSER, virtual regulators are not necessarily linked to immediate homeostatic deviations.

This leads to a crucial extension: the system acquires the ability to construct *second-order regulators*—goals that are not limited to achieving particular states, but can also modify the system’s own regulatory mechanisms.

#### 3.14.8 From adaptation to behavioral intelligence

As a result, the space of behavior expands beyond immediate adaptation.

The system is no longer restricted to restoring predefined variables, but can:

- define new preferred states,
- evaluate their attainability,
- and reorganize its own regulatory structure accordingly.

This transition provides the foundation for *proactive, goal-directed behavior*, and thus for what we term behavioral intelligence.

#### 3.14.9 Biological implementation of Ω^8^

Within this framework, the power of intelligence increases with the number of hierarchical levels. The question is how evolution achieves this.

There are two principal ways to increase the expressive capacity of such systems:

- increasing depth (adding layers),
- increasing width (expanding connections).

According to the universal approximation theorem (Cybenko, 1989), these approaches are functionally equivalent. Current ML mainstream follows the first path, but biological evolution appears to have followed the second path.

In mammals, the neocortex consists of only six morphological layers – a point worth qualifying: these cytoarchitectural layers should not be equated with the functional processing stages of artificial neural networks. Due to extensive recurrent and subcortical connectivity, the number of effective computational steps may be substantially larger. Instead of increasing morphological depth, evolution expanded the surface area, leading to the formation of cortical folds (gyri). This suggests that a shallow architecture was sufficient for biological intelligence.

The neocortex has a modular structure composed of *cortical columns* (~10^4^ neurons), with dense internal and relatively sparse external connections.

Columns receive input from the *thalamus* and send outputs back to the thalamus and basal ganglia. This supports the view, originally proposed by Vernon Mountcastle (1997), that columns function as basic computational units.

Following Jeff Hawkins (Hawkins et al., 2017), each column can be interpreted as a predictive unit. This aligns with Hierarchical Predictive Theory (HPT) and has been recently implemented in mathematical models (Pivovarov and Shumsky, 2022, 2025).

Evolution increases intelligence primarily by replicating such modules rather than deepening them.

Hierarchical structure arises through connections between columns:

- lower levels interact with sensorimotor systems,
- higher levels interact with other columns.

Thus, hierarchies of arbitrary depth can be constructed from repeated shallow modules.

#### 3.14.10 Ω^8^ emergence in evolution

From machine learning we know, that big hierarchical network requires:

- long learning periods
- safe developmental conditions (while intelligence not fully formed)
- huge amount of processing with high metabolic cost

This comparison concerns the architectural requirements for hierarchical prediction – depth, width, and extended learning time – not the specific learning algorithm. Biological learning mechanisms likely differ substantially from *backpropagation* and are generally more sample-efficient (Zador et al., 2023).

Within TEVSER, this is consistent with the emergence of Ω^8^ in *mammals*:

- extended development period (parental care, milk feeding),
- warm-blooded metabolism (much faster neuron processing)

Although early mammals appeared ~225 million years ago, this level likely matured in *placental mammals* (~70 million years ago).

From this point the neocortex becomes dominant:

- large surface area (gyri),
- high neuron count,
- modular structure.

#### 3.14.11 How decisions are made

Having introduced hierarchical prediction and goal-directed behavior, it is now necessary to clarify how decisions emerge from the interaction of multiple levels of self-representation.

Each new level of self-representation builds on earlier ones and operates jointly with them. Thus, although intelligence enables prediction and planning, it functions on top of, rather than instead of, lower-level mechanisms.

As a result, decision-making emerges from the entire ensemble of self-representations Ω^2^–Ω^8^.

Functionally, this can be viewed as a *distributed voting process*. Earlier systems (e.g., sensations, internal states, and reinforcement-learning signals processed via the basal ganglia) already contribute to action selection. The emergence of intelligence adds new inputs—predictions and evaluations of possible scenarios—which enter the same selection process.

The basal ganglia integrate these heterogeneous signals and determine the resulting action. Consequently, behavior reflects a combination of:

- emotional drives,
- conditioned responses,
- and higher-level reasoning.

This explains why decisions often contain both irrational and rational components, which are not separate processes but parts of a single integrated system.

#### 3.14.12 Who makes the decision

Different levels of self-representation operate at different speeds.

Lower (evolutionarily older) levels are optimized for rapid responses and act almost instantly. Higher levels, such as hierarchical intelligence, require more time, as they involve evaluating possible future scenarios.

As a result:

- fast decisions are often made by lower levels,
- while slower, deliberative processes involve higher levels.

This asymmetry is consistent with experimental findings (Libet et al., 1983; Bode and Haynes, 2009), which show that conscious awareness of a decision may arise 200–500 ms after its initiation.

Importantly, this does not imply that decisions are made “someone else.” Decisions are produced by the organism’s own control system—its self-representation.

However, not all layers of this system are always engaged. In particular, higher levels (Ω^6^–Ω^8^), associated with conscious evaluation, may be bypassed in fast or routine actions.

#### 3.14.13 Self-representation level Ω^8^

Conditioned reflexes, reinforcement learning, and hierarchical intelligence represent successive stages in the evolution of a single self-representation that progressively extends from the present into the past and future.

The key distinction between Ω^7^ and Ω^8^ lies not only in the prediction horizon, but in the explicitness of representation:

- Ω^7^: future scenarios are evaluated implicitly, and action selection is largely automatic, based on learned value functions.
- Ω^8^: the system constructs explicit, consciously accessible models of possible futures, enabling deliberate comparison and choice.

At this level, decision-making shifts from selecting individual actions to evaluating trajectories. The system considers sequences of states {*ω*_0_, …, ω_N_} and corresponding action chains {α_i_ → … → α_k_}, selecting those that maximize expected value:


$$\left\{\alpha_{i}\to \alpha_{j}\to \ldots \to \alpha_{k}\}=\text{argmax}\;Q^{’}(\omega \right)$$

$$\Omega^{8}⊃Q^{’}(\omega_{i})$$

Here Q’(ω) denotes a trajectory-level value function—distinct from the state-value function Q(ω) at Ω^7^—which evaluates entire sequences of states rather than individual states.

Each state ω_i_ includes both the system itself and relevant objects O_i_ in the environment, together with their possible dynamics. Thus, self-representation at Ω^8^ integrates:

- the agent,
- the environment,
- and their joint evolution over time.

However, physical space is not the only domain relevant to behavior. Organisms operate within additional meta-level spaces—such as social, symbolic, and abstract domains—each governed by its own regularities.

The same evolutionary logic implies that self-representation must eventually extend into these domains as well.

### 3.15 Mirroring and Ω^9^

#### 3.15.1 Survival in groups

The evolution of self-representation is driven by natural selection under increasing competition. As populations grow, competition intensifies, leading not only to interspecies competition but also to new forms of organization.

One of the most effective strategies that emerged is *group behavior*.

Living in groups increases survival probability:

- detection of predators scales with group size,
- while the risk for each individual decreases.

For predators, cooperation enables hunting larger prey and increases efficiency.

However, group living also introduces internal competition:

- for food and resources,
- for dominance and status,
- leading to the emergence of social strategies such as signaling, alliances, and conflict avoidance.

#### 3.15.2 Emergence of Ω^9^

These pressures lead to a new level of self-representation—Ω^9^, in which the agent represents not only itself, but also *other organisms of the same species*, denoted C_i_.

Crucially, these entities differ fundamentally from ordinary objects O_i_:

- unfamiliar objects are typically treated as potential threats,
- whereas conspecifics C_i_ serve as reference points for coordinated behavior.

Behavior is no longer based solely on the environment, but on the *interaction between agents*.

#### 3.15.3 From objects to minds

At this level, it becomes insufficient to track only external movements. Survival requires:

- predicting the behavior of others,
- and inferring their internal states.

Thus, C_i_ is not merely an object, but a representation that includes (at least partially) the self-representation of another agent:


$$C_{i}\approx \Omega (O_{i})$$

This marks a fundamental transition: the system begins to represent *other minds*.

#### 3.15.4 Mirror neurons

From the perspective of self-representation, a special class of objects emerges—conspecifics C_i_, which require a distinct mode of processing.

This implies the existence of specialized neural mechanisms, often associated with *mirror neurons*, which respond both to an agent’s own actions and to the observed actions of others.

There is ongoing debate as to whether mirror neurons constitute a distinct class or arise through learning during development. Mirror neurons should be understood as one debated mechanism relevant to action mapping and social cognition, rather than as its sole basis; the neural underpinnings of social cognition are distributed and not reducible to any single cell type. However, two empirical observations support the existence of dedicated mechanisms:

- not all organisms at levels Ω^5^–Ω^8^ exhibit group behavior,
- but species that do exhibit it reliably acquire such behavior.

This suggests that evolution has selected neural architectures that support social processing.

In primates, these mechanisms are complemented by the *prefrontal cortex*, which plays a central role in modeling both others and oneself in a social context.

#### 3.15.5 Expression: facial signals and gestures

Group survival depends not only on recognizing others, but also on being recognized.

Since internal states (psyche, intentions) are not directly observable, evolution favors mechanisms for *externalizing them*.

Facial expressions and gestures serve precisely this function:

- they encode emotional and motivational states,
- and make them accessible to other agents.

The more accurately such signals reflect internal states, the more effectively group members can:

- interpret each other,
- coordinate actions,
- and avoid conflict.

Thus, Ω^9^ includes both:

- inference of others’ states,
- and expression of one’s own states.

#### 3.15.6 Self-representation level Ω^9^

At this level, self-representation expands to include conspecific agents:


$$\Omega^{6}⊃O_{i}$$

$$\exists \varphi :\;O_{i}\cup \Omega (O_{i})\to C_{i}$$

$$\Omega^{9}⊃C_{i}$$

Thus, the system represents not only objects and itself, but also other agents as carriers of internal states.

#### 3.15.7 Ω^9^ emergence in evolution

Group behavior likely began to emerge during the Paleozoic era, possibly as early as the *Devonian* (~400 million years ago), in response to increasing predation.

Over time, mechanisms supporting group coordination became evolutionarily stable and independently emerged across multiple lineages.

For example:

- in fish, advanced sensory systems appear in the *Triassic* (~250 million years ago),
- and fossil evidence from the *Cretaceous* (~100 million years ago) suggests the presence of large coordinated groups (shoaling behavior).

This indicates that Ω^9^ is not tied to a single lineage, but represents a general evolutionary solution to the challenges of social living.

As the system increasingly relies on modeling other agents and their behavior, it must also represent itself within this space of interactions. In this sense, the emergence of social interaction naturally leads to the emergence of self-consciousness.

### 3.16 Self-consciousness and Ω^10^

#### 3.16.1 Ontogenesis and social origin

We have traced the evolutionary emergence of multiple levels of self-representation. However, higher levels are not fully innate: they are formed during ontogenesis through social interaction.

This idea was articulated by Vygotsky (2005), who argued that higher mental functions arise from interaction with others and culture.

A note on the nature of Ω^9^ through Ω^11^: unlike earlier levels, these do not describe single functional transitions but increasingly complex clusters of partially dissociable capacities. These capacities – including body-ownership, agency, autobiographical self-model, perspective-taking, language, and abstract symbolic reasoning – emerged gradually and potentially in different lineages. They are neurologically dissociable both developmentally and following brain damage. The taxonomic anchors and temporal estimates offered below are illustrative hypotheses within the TEVSER framework, not empirical claims about discrete evolutionary milestones.

#### 3.16.2 Evolution in a social environment

With the emergence of group behavior (Ω^9^), survival increasingly depends on interactions with other agents.

Over time, the group itself becomes an independent actor, controlling resources and territory. As a result, competition shifts:

- from environment → to within the group.

For the agent, the behavior of conspecifics C_i_ becomes more important than most environmental objects.

This marks a transition from physical space to a new domain—the *space of social interactions*.

#### 3.16.3 Self-consciousness (“subject who”)

Self-consciousness emerges as a higher-order level of self-representation through interaction with other subjects.

Before this stage, the agent has a first-person perspective (“subject here and now”), but does not explicitly recognize itself as a subject. Through social interaction—being addressed, evaluated, and responded to—the system begins to *direct attention toward itself* and becomes aware of the fact of its own existence.

Thus emerges the *“subject who”*:

- at Ω^3^: the subject is localized in space (“here”),
- at Ω^6^: the subject is localized in time (“now”),
- at Ω^10^: the subject is localized among other subjects.

This process is supported by developmental evidence: self-consciousness in humans typically appears around age ~3, together with the use of “I.”

The same idea appears in:

- Rubinstein (1940)—the subject is not given a priori, but emerges through activity, gradually separating itself from its immediate relations with the surrounding world.,
- Cooley (1902)—self emerges as a reflection of social feedback: individuals perceive themselves through the imagined perspective of others, effectively using social interaction as a “mirror.”

From this point, the subject is anchored in three fundamental domains: space (“here”), time (“now”), and social relations (“who”).

#### 3.16.4 Self as a social construct

Self-consciousness can be understood as a *socially constructed layer of subjectivity*.

As noted by Bing Song (2021), different cultures conceptualize the self differently:

- in Western traditions—as an individual entity,
- in Eastern traditions—as a node in a network of relationships.

Within TEVSER, this reflects the fact that Ω^10^ represents the self relative to other agents, rather than as an isolated entity.

#### 3.16.5 Self-consciousness as a key to others

One possible function of self-consciousness is improving prediction of others.

According to *social projection theory* (Allport, 1924), individuals tend to assume that others behave similarly to themselves.

Thus, modeling one’s own behavior provides a template for modeling others, improving adaptation in social environments.

#### 3.16.6 Awareness (sustained self-awareness)

Awareness, in this context, can be understood as a sustained form of self-consciousness—continuous self-directed attention to one’s own state. This usage differs from broader uses of the term “awareness,” which may refer to general perceptual or cognitive access to information.

Since attention (Ω^5^) is primarily oriented toward the external world, redirecting it inward requires effort. This aligns with the idea that conscious awareness requires effort and must be actively maintained (cf. Merab Mamardashvili).

#### 3.16.7 Self-representation level Ω^10^

At this level, self-representation includes a model of the self as an object of attention:


$$\exists \rho_{j}:\rho_{j}\;⊃\varphi \;(\Omega^{6})$$

$$\Omega^{10}⊃\rho_{j}$$

Thus, the system constructs a representation of itself based on:

- its current internal state (Ω^6^),
- and ongoing processes (Ω^7^–Ω^9^).

Importantly, this is not a complete model of the entire system, but a partial, attention-mediated representation of the self.

#### 3.16.8 Emergence in evolution

Self-consciousness likely begins to emerge in *primates*, where social complexity becomes critical.

Key factors:

- prolonged dependency of offspring,
- stable social bonds,
- increasing group complexity (*Miocene–Pliocene*, ~23–3 million years ago).

Neurobiologically, this is associated with the development of the *prefrontal cortex*, which supports:

- modeling of self and others,
- planning and social reasoning.

The size of the neocortex correlates with group size (Dunbar, 2009), suggesting that brain expansion is driven by social demands.

During ontogenesis, the prefrontal cortex matures slowly, reaching full development only in early adulthood (Kolk and Rakic, 2022).

#### 3.16.9 Relation to language

Self-consciousness likely predates language, but is significantly amplified by it.

Language provides symbolic tools (e.g., “I,” “you”) that make self-representation explicit. However, these symbols reflect already formed distinctions rather than creating them from scratch.

### 3.17 Abstract intellect and Ω^11^

Abstract intellect emerges as a further extension of self-representation beyond behavioral intelligence (Ω^8^) and social self-consciousness (Ω^10^). It is characterized by the ability to operate not only on objects and actions, but on *symbols and abstract representations*.

#### 3.17.1 Tools as extension of self

One of the earliest steps toward abstract intellect is *tool use*.

A tool transforms an external object Z_i_ into part of the operational system S. Although its representation O_i_ = *φ*(Z_i_) already exists at Ω^6^, at higher levels the system begins to treat this object as an *extension of itself*.

A familiar example is driving: with sufficient experience, the car becomes incorporated into self-representation, extending the “subject here” (Ω^3^) into the surrounding space. The effective spatial domain of the self expands with speed and action.

Tool use is not unique to *Homo habilis* (~2.5 million years ago) – it has been documented in non-human species including corvids and non-human primates, suggesting independent emergence in multiple lineages. Within TEVSER, the claim is not about when tool use first appeared in evolutionary history, but about the functional extension of self-representation it implies: the capacity to model objects as extensions of one’s own agency in relation to the world.

#### 3.17.2 Learning from others

With the emergence of Ω^9^ and Ω^10^, the agent can not only observe others but *learn from their experience*.

This requires:

- representing another agent’s actions,
- mapping them onto one’s own self-representation,
- and distinguishing between “self” and “other.”

Thus, social learning depends on both:

- representation of others (Ω^9^),
- and self-consciousness (Ω^10^).

The ability to learn tool use from others may therefore serve as an indicator of self-consciousness.

#### 3.17.3 Language and symbolic space

The key transition to Ω^11^ is the emergence of *language*.

The origins of language remain debated, with hypotheses ranging from gestural and vocal coordination to social bonding and symbolic cognition. Within TEVSER, regardless of its precise origins, language leads to a fundamental transformation: the introduction of a *symbolic space W*, in which objects, events, and internal states are represented by symbols.

Language amplifies earlier structures:

- objects differentiated at Ω^6^ receive names,
- behavioral models at Ω^8^ become expressible,
- social representations at Ω^10^ become communicable.

Crucially, symbols can be manipulated *independently of direct perception*. Thus, self-representation gains a new dimension: operating within an internal symbolic domain.

At the same time, this domain remains grounded:

- symbols map to internal representations, not directly to the external world,
- and can be projected back to lower levels when needed.

Language therefore acts as both:

- an accelerator of intelligence,
- and a multiplier of representational depth.

#### 3.17.4 Knowledge transfer

Language enables a qualitatively new form of learning: *learning from others’ reasoning* (Bennett, 2023).

The progression of learning can be summarized as:

- Ω^7^: learning from direct experience,
- Ω^8^: learning from internal simulation,
- Ω^10^: learning from observing others,
- Ω^11^: learning from others’ descriptions of their internal models.

This allows knowledge to:

- be transmitted across individuals,
- persist across generations,
- and be reused without direct experience.

Thus, language transforms individual intelligence into collective, cumulative knowledge.

#### 3.17.5 Self-representation level Ω^11^

At this level, self-representation includes a symbolic meta-space W:


$$\Omega^{6}⊃O_{i}$$

$$\exists \varphi :\;O_{i}\to w_{i},\;\;w_{i}\subset W$$

$$\Omega^{11}⊃W$$

The space W is not isomorphic to the external world Z, but rather a transformation of internal representations (Ω^6^–Ω^8^).

Thus:

- every external object Z_i_ can be mapped to O_i_,
- and some Oi can be mapped to symbols w_i_,
- but not all internal representations require symbolic encoding.

Importantly, W is self-generative: new symbols can be constructed from existing ones, forming increasingly abstract structures, some of which may not correspond directly to physical reality.

#### 3.17.6 Emergence in evolution

Language and symbolic cognition emerged relatively recently in evolutionary terms—approximately 150–100,000 years ago. Abstract cognition predates writing by a wide margin: formal written language and knowledge systems (~2–3 thousand years ago) served as powerful externalization and amplification mechanisms, enabling cumulative transmission of knowledge across generations.

Thus, Ω^11^ represents the culmination of the evolutionary trajectory of self-representation: a system capable of operating on abstract symbols, constructing models of arbitrary complexity, and transmitting them across individuals and generations

## 4 Testable hypothesis

Consciousness and internal self-representations are not directly observable variables and cannot be measured in a straightforward manner. Current experimental approaches rely on indirect proxies (e.g., neural dynamics, behavioral outputs, and perturbational responses), which are inherently limited and underdetermined. Despite these constraints, a viable theory must generate *falsifiable and operationalizable predictions*. The TEVSER framework satisfies this requirement by specifying a set of testable hypotheses linking hierarchical regulation, neural organization, and observable system dynamics.

### 4.1 H1: hierarchical organization of self-representations

*Hypothesis*

We hypothesize that increasing complexity of biological regulation is associated with a progressively more pronounced hierarchical organization of self-representations, reflected in multi-level neural dynamics operating at different temporal scales. Relevant TEVSER levels: Ω^2^ through Ω^11^.

*Experimental Paradigm (fMRI)*

Participants perform tasks requiring different levels of regulation:

- simple sensorimotor task,
- context-dependent task,
- high-level problem-solving task.

*Expected Results*

Higher-level tasks recruit larger-scale and more integrated brain networks, with increased processing time and broader inter-network connectivity.

*Support for TEVSER*

A systematic transition from local, fast dynamics to distributed, slower, and integrative dynamics across task levels.

*Falsification*

No differentiation in neural organization across levels of task complexity.

### 4.2 H2: integration of regulatory processes and conscious state

*Hypothesis*

We hypothesize that the level of consciousness is determined by the degree of integration between regulatory processes rather than by overall neural activity alone. Relevant TEVSER levels: Ω^5^ through Ω^10^.

*Experimental Paradigm (EEG / TMS–EEG)*

Comparison of brain dynamics across wakefulness, sleep, anesthesia, and disorders of consciousness, including perturbational paradigms.

*Expected Results*

Conscious states exhibit globally integrated, complex dynamics, whereas unconscious states show local, fragmented responses.

*Support for TEVSER*

Consciousness correlates more strongly with inter-level integration than with global activation.

*Falsification*

Conscious states persist despite a breakdown of large-scale integration.

### 4.3 H3: selective disruption of higher-order regulation

*Hypothesis*

We hypothesize that higher-order regulatory processes can be selectively disrupted, leading to loss of subjective access and goal-directed control while preserving lower-level regulatory and reactive functions. Relevant TEVSER levels: Ω^0^ through Ω^4^ (preserved) vs. Ω^5^ through Ω^10^ (disrupted).

*Experimental Paradigm (fMRI)*

Studies of patients with fronto-parietal damage, disorders of consciousness, or pharmacological suppression.

*Expected Results*

Preserved sensory and subcortical responses alongside reduced activity in fronto-parietal and integrative networks.

*Support for TEVSER*

Dissociation between intact basic regulation and impaired conscious access.

*Falsification*

Loss of subjective access is always accompanied by breakdown of basic regulatory processes.

### 4.4 H4: evolutionary gradient of self-representation

*Hypothesis*

We hypothesize that the complexity of self-representations increases along evolutionary gradients, in parallel with increasing neural complexity, behavioral flexibility, and predictive horizon. Relevant TEVSER levels: Ω^0^ through Ω^11^.

*Experimental Paradigm (EEG / Invasive Electrophysiology / Comparative Imaging)*

Cross-species comparison (e.g., insects, cephalopods, mammals) using prediction, violation, and multi-step decision tasks.

*Expected Results*

More complex systems exhibit longer-lasting, recurrent, and integrated neural dynamics reflecting extended temporal modeling.

*Support for TEVSER*

A consistent relationship between regulatory complexity and depth of predictive dynamics.

*Falsification*

Advanced predictive dynamics occur in systems lacking corresponding regulatory architecture.

### 4.5 H5: learning as expansion of self-representation

*Hypothesis*

We hypothesize that learning increases the depth and integration of self-representations by expanding the system’s predictive horizon and internal modeling capacity. Relevant TEVSER levels: Ω^7^ through Ω^8^.

*Experimental Paradigm (EEG / fMRI)*

Longitudinal training studies (motor, interoceptive, or cognitive learning).

*Expected Results*

Reduction in prediction error signals, increased late components associated with self-monitoring, and increased functional connectivity between control and valuation networks.

*Support for TEVSER*

Learning enhances internal modeling rather than merely strengthening stimulus–response mappings.

*Falsification*

Complex learning occurs without changes in predictive or self-monitoring processes.

### 4.6 H6: predictive modeling of regulatory consequences

*Hypothesis*

We hypothesize that self-representations encode predictions about the regulatory consequences of actions, and that the accuracy of these predictions determines both subjective agency and behavioral efficiency. Relevant TEVSER levels: Ω^5^ through Ω^8^.

*Experimental Paradigm (EEG / fMRI)*

Action–outcome tasks with manipulations of feedback accuracy and agency (e.g., altered feedback, delayed outcomes, loss of control).

*Expected Results*

Prediction errors are reflected in error-related neural responses and increased activity in self-monitoring networks; agency judgments track prediction accuracy.

*Support for TEVSER*

Neural and behavioral responses reflect violations of self-generated predictions rather than mere stimulus unpredictability.

*Falsification*

Agency and performance are independent of action–outcome prediction accuracy.

### 4.7 H7: stereotyped spatial organization of ganglia

*Hypothesis*

We hypothesize that individual ganglia across Bilateria exhibit a species-specific, spatially stereotyped organization, in which neuron positions, neurite trajectories, and input–output mappings are conserved and functionally differentiated. Relevant TEVSER level: Ω^2^. Within TEVSER, such ganglia constitute structurally fixed regulatory modules that serve as building blocks for the hierarchical self-representations described in H1.

*Prediction (Scalability*)

This modular organization is preserved and extended across scales, such that higher-order brain regions (including cortex) are composed of integrated assemblies of structurally constrained, functionally specialized modules.

*Experimental Paradigm (Connectomics / Imaging)*

- Cross-individual comparison of ganglia structure (connectomics).
- Cross-scale analysis of modular organization in larger brain regions (fMRI / mesoscale connectivity).

*Expected Results*

- High structural reproducibility of ganglia across individuals.
- Consistent mapping between structure and function.
- Presence of modular, hierarchically organized architecture at larger scales.

*Support for TEVSER*

Stable, non-random modular organization that scales into hierarchical networks.

*Falsification*

High structural variability, lack of structure–function mapping, or absence of modular organization at larger scales.

## 5 Discussion

In this work, we proposed TEVSER as a theory of evolving self-representations, describing how increasingly complex forms of regulation give rise to psyche, consciousness, and intelligence.

The central idea is that a living system can be understood as a hierarchy of self-representations *Ω*, emerging as control structures that regulate behavior and maintain homeostasis.

Thus, consciousness and intelligence are not single entities, but structured layers of self-representation, emerging through evolution.

TEVSER does not aim to provide a complete mechanistic account of all levels, but rather a unifying functional framework linking them. The proposed levels Ω^0^–Ω^11^ should be understood as idealized functional regimes rather than strictly discrete biological stages.

### 5.1 Relation to existing theories

TEVSER is consistent with, and integrates, several major theories of consciousness:

- Predictive coding / active inference (Karl Friston): TEVSER extends this framework by embedding prediction within a hierarchy of evolving self-representations (3.14.1)
- Theory of Functional Systems (Pyotr Anokhin): the notion of a goal as a “virtual regulator” generalizes the acceptor of action result (3.14.5)
- Global Workspace Theory (Baars, 1988): corresponds to mechanisms of attention and selective access at Ω^5^.
- Higher-Order Theories: relate to Ω^10^, where the system represents itself as an object.
- Integrated Information Theory: shares the idea of irreducibility, but TEVSER provides a constructive, evolutionary account of how such integration emerges.

Rather than competing with these theories, TEVSER provides a unifying framework that situates them at different levels of self-representation.

#### 5.1.1 Relation to integrated information theory

TEVSER shows a close conceptual alignment with Integrated Information Theory (IIT), proposed by Tononi (2004), while offering a complementary, mechanistic perspective.

IIT adopts a *top-down approach*, starting from phenomenology and postulating that conscious experience exists and is characterized by:

- structured information,
- and its irreducible integration.

From this perspective, a physical system is conscious to the extent that it generates integrated information, quantified by the measure *Φ*.

##### 5.1.1.1 From integration to mechanism

TEVSER can be interpreted as providing a constructive account of how such integrated structures arise.

In TEVSER, self-representation Ω:

- exists only at the level of the system as a whole,
- is not reducible to individual components,
- and therefore satisfies the requirement of integration in the IIT sense.

Moreover, the hierarchical structure Ω^1^–Ω^11^ describes a progressive increase in complexity of self-representation, which can be viewed as qualitatively corresponding to increasing levels of Φ.

##### 5.1.1.2 Key difference: class of systems

A central difference lies in the specification of systems capable of consciousness.

IIT does not restrict the class of systems in which consciousness may arise, implying that any system with sufficient integrated information may possess some degree of experience.

In contrast, TEVSER proposes that consciousness emerges specifically in systems that:

- maintain dynamic homeostasis,
- possess self-representation,
- and evolve under environmental pressure.

Thus, consciousness is not a generic property of information integration, but a feature of self-regulating systems.

##### 5.1.1.3 Key difference: emergence

IIT characterizes the properties of conscious systems but does not specify a concrete mechanism for their emergence.

TEVSER, in contrast, describes:

- the evolutionary pathway,
- the functional role,
- and the mechanism by which self-representation—and thus consciousness—arises.

##### 5.1.1.4 Summary

In this sense, TEVSER can be viewed as:

- consistent with IIT at the level of phenomenological constraints,
- but extending it by providing a mechanistic and evolutionary account of how integrated conscious structures emerge.

### 5.2 Addressing the hard problem of consciousness

Following Chalmers (1995), any theory of consciousness must explain how subjective experience—our inner phenomenal world—arises from physical processes in the brain.

Within TEVSER, this problem can be reformulated in terms of the emergence and structure of self-representation.

#### 5.2.1 From representation to self-representation

Any physical system can be described by an external observer through a model. However, such a model is not intrinsic to the system itself.

With the emergence of the first control loop, a fundamentally new phenomenon appears: a *model embedded within the system*—self-representation Ω^1^.

This representation:

- does not exist in any single component,
- arises from the interaction of many elements,
- and begins to regulate the system.

Thus, self-representation is not an external description, but an emergent control structure generated by the system itself.

#### 5.2.2 Emergence of subjective experience

At the level Ω^2^, signals become differentiated, giving rise to a primitive subjective domain.

These differentiated internal states already constitute a minimal phenomenological world: the system distinguishes between different internal conditions corresponding to different inputs.

Does this differentiation already include the *full richness* of phenomenological experience that we know from our own subjective world? No – full qualia, in the sense of temporally extended, spatially anchored, and memory-integrated experience, emerge at Ω^6^. However, organisms with Ω^2^ are not without inner experience: their differentiated regulatory states constitute proto-qualia – the minimal form of phenomenological content in TEVSER. Organisms with only Ω^0^ or Ω^1^ process signals without such a differentiated internal domain: there is regulatory activity, but no phenomenological content in the TEVSER sense.

This is consistent with the central ontological position of TEVSER:

qualia are not a product that emerges from self-representation – they are what self-representation is, as experienced from within the system. Phenomenology is not the outcome of function but its inner side: what function is for the system itself, not for an observer describing it from outside. At *Ω*^2^, this inner side is minimal but already present.

At higher levels:

- Ω^3^: introduces spatial perspective (“subject here”),
- Ω^5^: introduces attention,
- Ω^6^: introduces temporal presence (“subject now”).

Together, these levels give rise to conscious experience as a structured internal world.

#### 5.2.3 Why the hard problem arises

The hard problem arises because subjective experience is perceived as *immaterial and directly given*, while the brain is a *material system*.

This creates the apparent paradox:

*how can a material structure generate an immaterial conscious experience?*

From the perspective of TEVSER, this paradox results from a confusion between levels of organization.

Neurons, synapses, and their signals are material processes. However, self-representation Ω is not identical to any individual neuron or signal. It is generated by their dynamics, but exists only as a pattern of organization of the system as a whole.

Thus, self-representation is:

- grounded in material interactions,
- yet not reducible to any individual material component.

It is therefore natural that subjective experience appears “non-material”: what is experienced is not the physical substrate itself, but the higher-order dynamic structure it generates.

TEVSER describes this structure as existing in a meta-space *Ψ*—a space of dynamically organized control patterns emerging from neural interactions.

This meta-space is not separate from the physical world; it is a higher level of physical organization, analogous to how a living cell is more than any individual molecule.

The analogy with the living cell is intended to illustrate level-of-organization emergence: properties exist at the level of the organized whole that are not present in – and cannot be usefully predicted from – the properties of individual components.

We do not claim that this establishes ontological irreducibility of phenomenal experience in the sense intended by David Chalmers. Rather, as argued above, TEVSER identifies subjective experience *with the self-representational organization itself*, rather than treating it as a separate property that requires additional explanation. This is *descriptive level of organization*, not a separate substance.

Thus, the transition from “material brain” to “immaterial consciousness” is not a jump beyond physics, but a metasystem transition (Turchin, 1993): material interactions give rise to a higher-order self-representation;

subjective experience is not something separately produced by this self-representation, but is this self-representation itself, as it exists from within the system.

#### 5.2.4 Where is the “I”?

Within TEVSER, the “I” is not a fixed entity, but a dynamic center within the hierarchy of self-representations.

The full system corresponds to the entire stack Ω^0^–Ω^11^, but the experienced “I” is the current center of processing, typically associated with:

- Ω^3^: spatial localization (“here”),
- Ω^6^: temporal presence (“now”),
- Ω^10^: social self (“who”).

This center is not static; it shifts depending on context and task.

#### 5.2.5 Context-dependent localization of the “I”

In sensorimotor tasks requiring rapid interaction with the environment (e.g., sports or skilled movement), the center of experience is primarily localized at the levels Ω^3^–Ω^6^.

In such situations:

- attention is focused on external objects and immediate actions,
- behavioral intelligence (Ω^7^–Ω^8^) operates in the background,
- and tools (e.g., a racket or vehicle) may become incorporated into the operational self-representation.

As a result, the experienced “I” is centered on the “subject here and now,” without explicit self-reflection.

In contrast, in social contexts (e.g., public speaking or interaction with an audience), the center shifts toward Ω^9^–Ω^10^.

Here:

- the system continuously models other agents,
- evaluates their reactions,
- and positions itself relative to them.

In this case, the experienced “I” corresponds to the “subject who,” defined through interaction with others.

In tasks involving symbolic reasoning (e.g., writing or abstract thinking), the center may shift further toward Ω^11^.

Under these conditions:

- attention is directed toward symbolic structures,
- lower-level sensations may become attenuated,
- and the system operates primarily within the symbolic space.

This corresponds to a state of deep cognitive immersion, in which the experienced “I” is effectively embedded within the symbolic domain.

#### 5.2.6 Dynamic nature of the self

These examples illustrate that the “I” is not a fixed locus, but a context-dependent dynamic attractor within the hierarchy of self-representations.

Its position continuously shifts depending on:

- task demands,
- environmental context,
- and internal goals.

This dynamic organization explains the fluid and situational nature of subjective experience.

#### 5.2.7 Do cells and animals have consciousness?

Within TEVSER, consciousness is not a binary property but a *graded phenomenon* corresponding to different levels of self-representation:

- Cells: Ω^1^ only → no psyche or consciousness
- Animals: Ω^2^ → primitive subjective world
- Vertebrates: Ω^3^–Ω^6^ → consciousness (“here and now”)
- Humans: Ω^10^–Ω^11^ → self-consciousness and abstract thought

Thus, the question “do animals have consciousness?” is replaced by a more precise question:

which levels of self-representation are present in a given organism?

### 5.3 Addressing the problem of qualia

One of the central challenges in theories of consciousness concerns the nature of *qualia*—the subjective, qualitative aspects of experience, such as the “redness” of red or the “painfulness” of pain. Qualia are typically understood as the full subjective content of experience: the complex, unified inner perception that we directly experience.

For example, when one looks at a computer, the experience is not limited to a set of isolated features. It includes:

- its visual properties (shape, color, brightness),
- its interaction possibilities (keys, mouse, screen elements),
- its functional associations (writing, reading, programming),
- and all the tasks and intentions connected with it.

All these aspects are perceived both separately and as a unified whole. This raises the question: how is such a unified experience formed, and what is its function?

#### 5.3.1 Qualia as structured situational awareness

We propose that qualia can be understood as the system’s moment-to-moment situational awareness—the full internal representation of the current situation, constructed in order to support decision-making.

This concept is closely related to the notion of *situational awareness*, often used in military contexts to describe a comprehensive understanding of:

- the environment,
- relevant objects,
- possible actions,
- and associated risks.

Within TEVSER, qualia correspond to an internal state that integrates:

- current perception,
- relevant objects,
- and memory traces associated with the current situation.

#### 5.3.2 Qualia and memory integration

The key role of memory in the formation of qualia is not the storage of information per se, but its selective activation in context.

Memory developed through evolution as a feature to help an agent to survive. Consider an animal in a natural environment.

When a deer steps into a clearing, it does not perceive only the immediate visual scene. Its perception is enriched by:

- remembered locations of food,
- known paths and escape routes,
- past encounters with predators,
- and recent traces such as smells or movement patterns.

Each element of the scene is connected to a network of past experiences. These connections can be thought of as latent taut strings, ready to activate additional layers of information when needed.

Thus, perception is not static. It is dynamically extended by memory into a richer representation of the current situation.

#### 5.3.3 Qualia as a unified field

Qualia are therefore not simply raw sensory inputs, nor are they abstract representations.

They are a unified experiential field, in which:

- multiple modalities are integrated,
- relevant memories are activated,
- and the current situation is represented in a form suitable for immediate action.

In this sense, qualia are:

The full immediate feeling of the situation at a given point in space–time, connected through memory to past events.

This definition preserves the phenomenological character of qualia while grounding them in the functional architecture of self-representation.

Importantly, this interpretation does not reduce qualia to their functional role. Within TEVSER, qualia are not a product that emerges from self-representation – they *are* what self-representation is, as experienced *from within* the system. Phenomenology is *not the outcome* of function but its inner side: what function *is for the system itself*, not for an observer describing it from outside.

#### 5.3.4 Function of qualia

From an evolutionary perspective, qualia are not incidental—they serve a critical function.

They provide the organism with:

- a compressed yet information-rich representation of the current situation,
- immediate access to relevant past experience,
- and a basis for rapid decision-making in dynamic environments.

Thus, qualia can be understood as the operational form of situational awareness, optimized for survival.

#### 5.3.5 Relation to the hard problem

The apparent “mystery” of qualia arises when qualitative experience is considered independently of the system that generates it.

Within TEVSER, subjective experience emerges progressively across the hierarchy – from proto-qualia at *Ω*^2^ to the full phenomenological world at Ω^6^, where the system integrates its internal states into a temporally and spatially coherent structure.

From this perspective, qualia are neither properties of the external world nor separate non-physical entities. Rather, they are how the system’s internal states are organized and experienced from within.

This provides a constructive approach to the hard problem: instead of asking how physical processes generate “qualitative experience,” the question becomes how systems construct differentiated internal state spaces that are experienced as qualitative.

It is important to state the ontological position of TEVSER precisely. The theory does not propose that self-representation Ω generates or causes subjective experience as a separate phenomenon. Rather, TEVSER identifies subjective experience *with the self-representational dynamics of the system, considered from within*.

There is no additional explanatory step from *Ω* to experience, because they *are* the same phenomenon described from two perspectives: the external third-person description of neural dynamics, and the internal first-person character of experience.

The apparent explanatory gap arises only if subjective experience and self-representation are assumed to be ontologically distinct entities. Those who maintain, with Chalmers (1995), that phenomenal properties are irreducible to any functional or organizational description will not accept this identification – and we acknowledge this represents a genuine divergence of ontological commitments, not a question resolvable within the present framework alone.

What TEVSER offers is a constructive, empirically grounded account of how the relevant self-representational structure arises, and a precise characterization of its organization. Whether this constitutes a “solution” to the hard problem depends on prior ontological commitments about the nature of phenomenal reality.

Within TEVSER, there is no subjective experience that falls outside *Ω* – every form of experience, from the most primitive nociceptive state to the richest conscious moment, corresponds to some level or configuration of self-representation. Pain, thirst, joy, and abstract thought are not separate phenomena requiring independent explanation: they are specific configurations of Ω at the relevant level of the hierarchy.

### 5.4 Consciousness and subjectivity in artificial agents

The rapid progress of artificial intelligence naturally raises the question of whether consciousness and subjectivity can emerge in artificial agents. This question is particularly relevant within TEVSER, which provides a constructive framework for analyzing such possibilities.

#### 5.4.1 Intelligence without consciousness

Modern large language models (LLMs) can be interpreted as partial implementations of high-level self-representation, approximately corresponding to *Ω*^11^, but detached from the lower levels.

These systems:

- operate within symbolic space (language),
- use attention mechanisms (analogous to Ω^5^) for prioritization,
- and perform prediction over sequences.

However, this constitutes *intelligence without subjectivity*.

The system processes information, but there is no integrated self-representation to which this processing belongs.

Even in multimodal systems, which incorporate images, audio, or video, the resulting models at best approximate fragments of:

- Ω^6^ (object representations),
- Ω^7^ (learning from feedback),
- Ω^8^ (hierarchical prediction).

Yet these remain disconnected representations, lacking a unifying self-representation responsible for maintaining homeostasis.

In this sense, current AI systems implement isolated layers of the Ω, rather than the full architecture.

#### 5.4.2 The role of embodiment and grounding

A fundamental requirement for a true artificial agent is *embodiment*—the ability to distinguish itself from the external world.

This may take the form of:

- a physical body, or
- a bounded presence within a virtual environment.

Such embodiment enables the core regulatory cycle:

*action→perception→prediction error→model update→action*

Without this loop, representations remain ungrounded.

In machine learning, this problem is commonly referred to as *grounding*—the need to connect symbolic representations to real-world objects and processes.

Within TEVSER, grounding corresponds to the integration of higher levels (Ω^10^–Ω^11^) with lower levels of self-representation.

#### 5.4.3 Is consciousness possible in artificial agents?

From the perspective of TEVSER, the emergence of consciousness in artificial systems is, in principle, possible.

Biological neurons themselves are simple computational units, operating at the level of basic signal processing. However, when organized into a system, they give rise to higher-order self-representations that regulate the system as a whole.

Artificial neurons are not fundamentally different in this respect. While individually simple, they can, in principle, be organized into architectures capable of generating higher-level self-representations.

Thus, consciousness is not tied to a specific biological substrate, but to the *organization of the system*. At the same time, the theory does not exclude the possibility that certain physical properties of substrates – including properties not yet fully characterized – may be necessary conditions for the relevant organizational dynamics.

#### 5.4.4 Do artificial agents need consciousness?

A further question concerns not possibility, but necessity.

If consciousness arises as part of a hierarchy of self-representations supporting homeostasis, it is not evident that artificial agents require the full stack of such representations in order to function effectively.

At the same time, the prospect of conscious artificial agents raises a set of fundamental concerns that are central to current discussions in AI safety:

- *Do we want* artificial systems to develop self-preserving goals?
- Will such agents remain safe for humans?
- Could such systems, if endowed with self-preserving goals and advanced forms of self-representation, compete with humans and potentially displace them in key domains or even as a dominant species?

These concerns are not merely speculative. They reflect a widespread intuition that the emergence of consciousness may be directly linked to autonomy, self-interest, and potentially uncontrolled behavior.

Within the TEVSER framework, these questions can be reformulated in a more precise way: the properties typically associated with “dangerous” artificial agents are not a direct consequence of intelligence or even consciousness per se, but of how self-representation is structured, how it is coupled to goals, and how regulatory mechanisms are defined.

This perspective suggests a more nuanced—and in fact *more* *optimistic*—view. In particular, TEVSER implies that the safety of artificial agents depends not on preventing the emergence of higher-level self-representations, but on the architecture of the regulatory hierarchy itself.

A detailed analysis of these implications, including potential design principles for safe artificial agents, follows directly from the proposed framework and will be developed in future work.

### 5.5 Implications and limitations

#### 5.5.1 Implications

The TEVSER framework provides a unified perspective on the emergence of psyche, consciousness, and intelligence as successive levels of self-representation in self-regulating systems.

This perspective has several important implications:

- It suggests that consciousness is not a binary property, but a *graded phenomenon* corresponding to different levels of self-representation.
- It provides a *constructive account* of subjective experience, linking phenomenology to underlying system dynamics.
- It reframes the hard problem of consciousness as a question of *levels of organization*, rather than a fundamental gap between physical and non-physical domains.
- It offers a *common framework* for integrating insights from neuroscience, psychology, and artificial intelligence.
- It suggests that intelligence and consciousness can, in principle, be implemented in non-biological systems, provided the appropriate architecture of self-representation is realized.

Taken together, these implications point toward a view of consciousness as an emergent property of hierarchically organized control systems.

#### 5.5.2 Limitations

At the same time, several important limitations of the present work should be acknowledged.

First, the theory is primarily conceptual and does not introduce new empirical data. It is based on the synthesis and reinterpretation of existing findings across multiple domains.

Second, although TEVSER proposes a hierarchy of self-representation levels, the mapping between these levels and specific neural structures or circuits remains approximate. The theory does not yet identify precise neural implementations for each level.

Third, the mechanisms underlying the emergence and interaction of self-representations are described at a functional level. The exact neural processes and signaling patterns that instantiate these dynamics remain to be specified.

Fourth, some of the proposed predictions may be challenging to test empirically with current methodologies, particularly those involving higher-level or distributed representations.

Fifth, the framework relies on a set of working definitions of key concepts such as self-representation, consciousness, and qualia. While these definitions are grounded in existing literature, alternative formulations may lead to different interpretations.

Sixth, the boundaries between *Ω*-levels are presented as discrete for clarity, whereas in biological systems these transitions are likely gradual and overlapping.

Finally, several extensions discussed in this work—including applications to artificial agents and social systems—remain hypothetical and require further theoretical and empirical development.

#### 5.5.3 Directions for future work

Importantly, these limitations should not be viewed solely as weaknesses of the framework, but as guides for future research.

They point toward:

- the need for empirical identification of neural correlates of Ω-levels,
- the development of computational models of self-representation,
- experimental validation of the proposed hierarchy,
- and further exploration of the framework in artificial and social systems.

A different paradigm of neural network operation will be required there. For example, the work of Eugene Izhikevich (Izhikevich and Edelman, 2008) demonstrates how complex patterns of network behavior can emerge in spiking neural networks, influencing the activity of individual neurons.

The primary function of the brain is to support the operation of our self-representation, whose ultimate goal is the regulation of homeostasis, as has been compellingly shown in recent work (Theriault et al., 2025).

In this sense, TEVSER provides not only a conceptual model, but also a *research program* for investigating the emergence of consciousness and intelligence.

## 6 Conclusion

TEVSER provides a unified framework for understanding the emergence of psyche, consciousness, and intelligence as stages in the evolution of self-representation. Consciousness is not treated as a singular phenomenon, but as a *structured and graded property* emerging from the organization of self-regulating systems.

A central ontological commitment of TEVSER is that subjective experience is not a product generated by self-representation, but its *inner side* – what self-representation *is*, as experienced *from within* the system.

On this view, phenomenal experience begins already at Ω^2^ in the form of proto-qualia, and progresses through increasingly rich forms – spatial perspective, attentional modulation, temporal depth – to the full phenomenological world at Ω^6^. Consciousness, in TEVSER, is *not a threshold but a progression*.

By distinguishing levels of organization and their functional roles, the theory offers:

- a constructive account of subjective experience,
- a resolution of the apparent paradox of the hard problem,
- and a bridge between biophysics, neuroscience, psychology, and artificial intelligence.

The framework further suggests that intelligence and consciousness can, in principle, be realized in artificial systems.

Importantly, the limitations of this framework should be viewed not as drawbacks, but as guides for future research.

## Data Availability

The original contributions presented in the study are included in the article/supplementary material, further inquiries can be directed to the corresponding author.
